# Supplementary material for: The impact of imputation quality on machine learning classifiers for datasets with missing values
Source: Commun Med (Lond). 2023 Oct 6;3:139. doi: 10.1038/s43856-023-00356-z (PMC10558448; doi:10.1038/s43856-023-00356-z)
Supplement: Supplementary file 1 — Supplementary Information [file 43856_2023_356_MOESM1_ESM.pdf]

Supplementary Information for The Impact of Imputation Quality on Machine Learning Classifiers for Datasets with Missing Values

Tolou Shadbahr, Michael Roberts, Jan Stanczuk, Julian Gilbey, Philip Teare, Sören Dittmer, Matthew Thorpe, Ramon Viñas Torné, Evis Sala, Pietro Lió, Mishal Patel, Jacobus Preller, AIX-COVNET Collaboration, James H. F. Rudd, Tuomas Mirtti, Antti Sakari Rannikko, John A. D. Aston, Jing Tang, Carola-Bibiane Schönlieb

Supplementary Notes

Comparison between MSE and Wasserstein distance as discrepancy scores

In Supplementary Figure 1 below, we show an example data distribution, created with the `make_classification` function, similar to the **Simulated (N)** dataset. We evaluate the MSE and Wasserstein Distance between true and imputed data for (b) a method that optimises for MSE and (c) using MICE. It is clear that optimising for MSE leads to a poor distribution reconstruction, however, MICE reflects the underlying distribution well. This simple example demonstrates how MSE is insufficient for assessing imputation quality, as although MICE is clearly preferable in this case, it has a poor MSE.

(a) True Data Distribution	(b) Imputation optimising mean square error	(c) Imputation with the MICE method
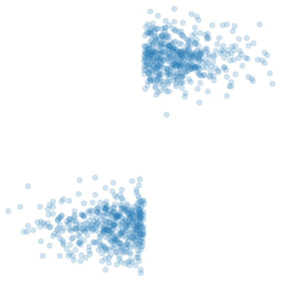	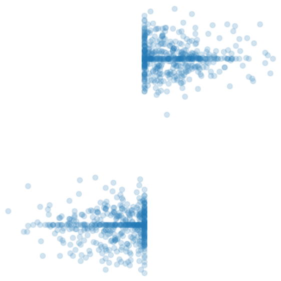	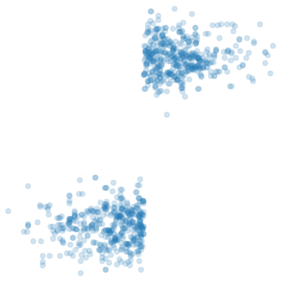
	Mean Square Error = **0.9074**<br>Wasserstein Distance = $\mathbf{2.68}\times10^{-4}$	Mean Square Error = **1.6533**<br>Wasserstein Distance = $\mathbf{1.55}\times10^{-4}$

Supplementary Figure 1. Comparing imputation for MSE and MICE. In (a) we see a simulated dataset, (b) shows the optimal imputation against the mean square error (MSE) and (c) shows the MICE imputation result. The MSE and Wasserstein distances are quoted for each.

Dataset Description and Preprocessing Details

All of the datasets used in this study are publicly available (upon reasonable request for **MIMIC-III** and **NHSX COVID-19**). Details for **Synthetic (N)** are included in the main paper. For the **MIMIC-III** and **Breast Cancer** datasets, we include some details below for how the **MIMIC-III** and **Breast Cancer** datasets were preprocessed for use in this study. The code for performing this preprocessing is also available in the codebase.

MIMIC-III. In preprocessing the MIMIC-III[1] dataset, we only considered information for the first ICU admission of patients, and restricted to patients who were over 15 years old, spent at least 3 days in the ICU in the data collection period and were admitted as ‘Emergency’ or ‘Urgent’ cases (21 812 patients). We extracted data on seven clinical variables: blood pressure (systolic, diastolic and mean), heart rate, oxygen saturation, respiratory rate and temperature; these were the only variables recorded for over 50% of the patients. In our preprocessing of the dataset, we only considered data from the first 10 days in the ICU (or the whole ICU stay if shorter), and excluded any patients who had fewer than 5 observations in any of aforementioned variables. (169 patients were excluded in this way, about 0.8% of the total, leaving 21 643 patients.) We then calculated the mean and standard deviation for each of these seven variables, giving a total of 14 numerical variables per patient (and no missing data). The outcome variable we use is the survival of patients in the 30 days after admission to the ICU. Our

preprocessing code is based on MIMIC-Extract[2] and is available at **[shared upon publication]**. Due to the size of the dataset (and the number of computations we ultimately perform), we then select one third of the patients randomly, resulting in a dataset with 7214 patients.

Breast Cancer. This dataset is derived from one collected at Memorial Sloan Kettering Cancer Center between April 2014 and March 2017[3]. The biopsy samples were collected prior to, during or after treatment from different primary or metastatic sites which leads to several samples for each patient. We only considered the first occurrence of the patients' ID. The dataset, consisting of 16 different features, is assembled using data stored in three different sub-datasets: `data_clinical_sample`, `data_clinical_patient` and `breast_msk_2018_clinical_data` from Razavi et al.[3]. Specifically, the features we consider are the 'Fraction Genome Altered' and 'Mutation Count', taken from `breast_msk_2018_clinical_data`, 'ER Status of the Primary', 'Invasive Carcinoma Diagnosis Age', 'Oncotree Code', 'PR Status of the Primary', 'Overall Primary Tumor Grade' and 'Stage At Diagnosis', taken from `data_clinical_sample` and 'Metastatic Disease at Last Follow-up', 'Metastatic Recurrence Time', 'M Stage', 'N Stage', 'T Stage', 'Overall Patient HER2 Status', 'Overall Patient HR Status' and 'Overall Patient Receptor Status' from `data_clinical_patient`. The features of this dataset are of several different types, 'ER Status of the Primary', 'Metastatic Disease at Last Follow-up', 'M Stage', 'Overall Patient HER2 Status', 'Overall Patient HR Status' and 'PR Status of the Primary' are binary; 'N Stage', 'T Stage', 'Stage At Diagnosis' and 'PR Status of the Primary' are ordinal; 'Oncotree Code' and 'Overall Patient Receptor Status' are multilevel categorical and 'Fraction Genome Altered', 'Invasive Carcinoma Diagnosis Age', 'Metastatic Recurrence Time' and 'Mutation Count' are numerical. The outcome variable we choose was 'Overall Survival Status' for the classification task. Moreover, features 'N Stage' and 'T Stage' are described with 14 and 15 different levels respectively where some of the levels only have one or two samples. To ensure features are meaningfully represented, we only consider the parent family of each level which results in only 4 different levels for each feature. All the ordinal variables are treated in the same manner as numeric variables if the imputation method was not capable of addressing the ordinal variables. The majority of categorical variables are binary, or have only two options (e.g. sex, death) and are simply encoded to zero and one and treated as numeric variables or binary (if the imputation method was capable of modelling the binary variables).

Synthetic (N,C). This is a synthetic dataset created using the `scikit-learn`[4] function `make_classification`, giving a dataset with 1000 samples, 20 informative features and 5 useless features. Of the informative features, 10 are left as continuous variables, 5 are converted into ordinal features with between 3 and 6 values, one is converted into a factor with four different values and the remaining 4 are converted into binary factors. The useless features are handled similarly: 3 are left as continuous variables, one is converted into an ordinal feature with 4 values and one is converted into a binary categorical variable. The code to recreate this dataset can be found in our repository, along with the resulting dataset.

Descriptions of the imputation methods

Below, we give a detailed summary of each imputation method used in this paper, in particular, we detail how the categorical variables are encoded for each method.

Mean Imputation is one of the simplest imputation methods, in which the missing values are replaced with the sample mean. This is a single imputation method, as there is no stochasticity in its computation. Mean imputation was performed using the `SimpleImputer` implementation in the Python package `scikit-learn`[4]. Mean imputation has no special treatment for the multi-level categorical variables, therefore we one-hot encode all the multi-level categorical features as part of the preprocessing.

MissForest is an iterative imputation method that employs a random forest (a non-parametric model) for non-linear modelling of mixed data types. Therefore, MissForest is applicable for numerical, categorical and ordinal data, whilst making few assumptions about the structure of the data[5,6]. MissForest imputation works by initially training a random forest using the observed (i.e. not missing) data values to predict the missing entries of a given variable. This procedure continues until either a maximum number of iterations is reached or the out-of-bag error increases[5,7]. MissForest imputation was performed using the Python package `missingpy`[8]. In our implementation, the multi-level categorical variables in **Breast Cancer** are specified to the algorithm.

Multivariate Imputation by Chained Equations (MICE), also known as 'Fully Conditional Specification'[9] or 'Sequential Regression Multivariate Imputation'[10], is an imputation method which iteratively imputes the missing values of each variable one at the time using a method based on the type of the corresponding variable (such as linear regression)[7,11,12]. In its initialisation, MICE sets the missing values using values derived from the observed values, for example, the mean of the feature

or a random observed value. Next, a model is fit which takes one feature as the output/target variable and all other features are used as input variables. This model is then applied to predict the target variable and those which correspond to the missing values are replaced. This process is repeated, updating all features in turn. This gives a dataset in which all missing values of the features have been updated once. This whole process can be repeated many times until the imputed values converge to within some error[13–15]. We perform our computation using the R package `mice`[16]. In our model, all the numerical variables are initially imputed using predictive mean matching (pmm)[16]. In the **Breast Cancer** dataset, there are variables with multi-level categorical values. These variables are one-hot encoded and imputed using logistic regression along with binary variables. Finally, the ordinal variables are coded with numeric values and imputed using a proportional odds model.

Generative Adversarial Imputation Networks (GAIN)[17] is an imputation method whose framework is based on generative adversarial networks[18]. This approach adversarially trains a pair of neural networks, first a generator whose purpose is to generate realistic samples from the development distribution and a discriminator which aims to identify whether a sample is from the development distribution. Training in this adversarial manner ensures that the generator creates more realistic samples as it tries to "fool" the discriminator. For the GAIN method, a binary mask is also provided to the generator function where the zeros correspond to the missing values and ones indicate an observed value. A "hint" matrix is generated from this mask for which some proportion of entries (decided by a parameter) are set to 0.5, i.e. for these entries we do not know if the value is observed or missing. Initially, all missing values are replaced by random noise sampled from a normal distribution. The generated dataset is input to the discriminator along with the hint matrix. The task of the discriminator is to predict the mask, i.e. identify both the observed values and imputed values[17,19]. For GAIN, the official implementation provided in the paper[17] is used. As GAIN cannot directly use multi-level categorical variables as input, in our implementation these are one-hot encoded and the ordinal features are replaced with numerical values before imputation with GAIN.

Missing Data Importance-Weighted Autoencoder (MIWAE) is an imputation method proposed by Mattei and Frellsen[20] which uses a deep latent variable model (DLVM)[21,22] for the imputation of the missing values in a given dataset. This method builds upon the importance-weighted autoencoder (IWAE)[23], which aims to maximise a lower bound of the log-likelihood of the observed data. The lower bound of IWAE is a k-sample importance weighting estimate of the log-likelihood and is a generalisation of the variational lower bound used in variational autoencoders[21] (which corresponds to the case of $k = 1$). In MIWAE, the lower bound of IWAE is further generalised to the case of incomplete data (and coincides with the IWAE bound in the case of complete data). During training, the missing data is replaced with zeros before being passed into the encoder network to obtain the latent codes. The codes are passed to the decoder network and the output is compared with the observed data (only in non-missing dimensions) to compute the aforementioned lower bound. Once the model is trained, multiple imputation is possible via *sampling importance resampling* (SIR) from the trained model. We used the official Python implementation[20] in our study. All the multi-level categorical variables are first one-hot encoded and the ordinal features are coded with numerical values before imputation with MIWAE.

Hyperparameter selection for imputation methods

For GAIN and MIWAE imputation methods, there are several hyperparameters which must be tuned for optimal performance. In our experimentation, we used the default parameters for both of these models after tuning of the imputation models and finding that the changes in the loss values and imputed values were nominal. Therefore, we found it unjustifiable to jointly optimise over the imputation and classification method's hyperparameters as this would exponentially increase the computational (and carbon) costs of our experiments without expected gains.

Descriptions of the classification methods

In the following, we briefly describe each of the classification methods used in this paper. Moreover, we detail the hyperparameters, including the ranges of values, that are tuned in the benchmarking exercise for obtaining the optimal performing classifier.

Logistic Regression. This is a simple and efficient classification method with high prediction performance for datasets that have linearly separable classes. This method use a logistic function $\frac{1}{1+e^{-(mx+b)}}$ to generate a binary output, where x is the input and m and b are learned[24]. We used the `scikit-learn` library implementation of the logistic regression classifier. The only hyperparameter to tune is the maximum number of iterations used, we search over $\{50, 100, 150, 200, 250\}$.

Random Forest. This classifier is one of the most popular and successful algorithms. It was proposed by Breiman[6] and involves creating an ensemble of randomised decision trees whose predictions are aggregated to obtain the final results. In training, for each tree m samples are randomly selected by bootstrapping. Then, this subset of the dataset is used to train a randomised tree. This procedure is repeated k times. The Random Forest is the aggregation of these k decision trees[25]. For our

study, we used the Random Forest implementation in open source `scikit-learn` library[4] in Python. The Random Forest can be applied to a variety of different prediction problems and few parameters need to be tuned. For the number of estimators, we tried 8 different values in the range $[20,90]$ with equal step width as 10. For the maximum depth, we limited our search to $\{3,4\}$. For the minimum sample split and the minimum samples in each leaf, we search over the set $\{2,3,4\}$. This results in 144 different hyperparameter combinations.

XGBoost. This method, proposed by Chen and Guestrin[26], is an efficient gradient tree boosting algorithm which builds a chain of 'weak learner' trees to give a high-performing classification or regression model. In this method, a base model is constructed by training an initial tree. Then, the second tree is obtained through combination with the initial tree. This procedure is repeated until the maximum number of trees is reached. These additive trees are selected based on a greedy algorithm, in each step adding the tree that most minimises the loss function. Therefore, the training of the model is in additive manner[26]. For the implementation of XGBoost, we used the official `xgboost` Python package provided in[26]. For the maximum depth of trees, we consider 3 different values $\{3,4,5\}$ and for the number of subsamples we selected among 6 different values in the range $[0.5,1]$ with equal step size 0.1. The number of trees are selected from 8 different values, in the range $[50,400]$ with equal step size 50. This results in 144 different hyperparameter combinations.

NGBoost. This recent method, proposed by Duan et al.[27], gives probabilistic predictions for the outcome variable y for input x. It assumes there is an underlying probability distribution $P_\theta(y|x)$ with a parametric form, described by $\theta(x)$. This method considers natural gradient boosting, by replacing the loss function with a scoring rule. This scoring rule, compares the estimated probability distribution to the observed data, by using a predicted probability distribution P and the observed value y (outcome). The proper scoring rule S returns the best score for the true distribution of the outcomes. The parameter that minimises the value of the scoring rule is obtained through natural gradient descent[28]. For the implementation of the NGBoost method, we used provided code on the official repository at `https://github.com/stanfordmlgroup/ngboost`. An NGBoost model has three key hyperparameters which can be tuned, namely the learning rate, the minibatch fraction and the number of estimators. For the learning rate we used 4 different values $\{0.0001,0.001,0.01,0.1\}$. For the minibatch fraction, we used 6 different values in the range $[0.5,1]$ with equal step size 0.1. The number of the estimator is selected from 8 different values in the range $[50,400]$ with equal step size 50. This results in 192 different hyperparameter combinations.

(Artificial) Neural Network (NN). The artificial neural network we employ is a multi-layer perceptron (MLP)[29] consisting of layers of neurons. Each neuron is assigned a value, based on a weighted combination of the values for neurons in the previous layer. Activation functions are employed to introduce non-linearities into the weighted sum calculations. The training of the NN is through backpropagation[30], where the weights of the edges between each neuron are adjusted to minimise the error between the true labels and output. We use the ReLu[31] activation function and the ADAM[32] optimiser method with binary cross-entropy as the loss function. We used the implementation in the open source `scikit-learn` library[4] in Python. An NN has three key hyperparameters which must be tuned, specifically, the initial learning rate for the optimiser, the number of hidden layers and the number of neurons in each hidden layer. The initial learning rate selected from 3 different values $\{0.001,0.01,0.1\}$, the number of the hidden layers is set to one of the 3 values $\{1,2,3\}$. 5 different values are considered for the number of the neurons in each hidden layer from the range $[20,100]$ with equal step size 20. This results in 45 different hyperparameter combinations.

Data partitioning and hyperparameter selection

We partition each dataset at two levels as shown in Supplementary Figure 2. In the first level, we randomly partition the dataset into three holdout sets, each consisting of one third of the samples. These are used for reporting the performance of each imputation method and classifier. Each holdout set has a complementary development set and at the second level of partitioning, we divide the development set into five non-overlapping cohorts. We use five-fold cross-validation on the development set to select the optimal hyperparameters for each combination of imputation method and classifier using the mean area under the receiver operating characteristic curve (AUC) over the five validation folds. In Table 1, we detail the hyperparameters combinations considered for each of the classifiers.

Description of the ANOVA analysis

In our multi-factor ANOVA analysis, for the **MIMIC-III** dataset we included the missingness rate of the development data and holdout data as separate factors to allow us to evaluate their individual effects. However, the **Breast Cancer** and **NHSX COVID-19** datasets are real datasets with their inherent missingness rates. Therefore, the missingness rate is introduced as a factor (i.e. categorical 25%, 50%, inherent) to the ANOVA model.

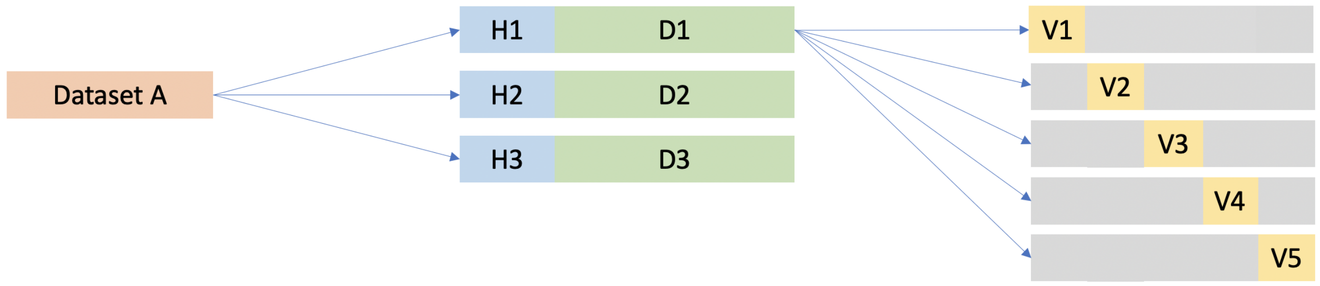

Supplementary Figure 2. A schematic illustrating the hierarchical dataset split. Key: H = Holdout, D = Development, V = Validation.

**Classifier (K)**	**Tuned Hyperparameters**
Random Forest (144)	Number of trees (8), maximum depth of trees (2), minimum samples needed for splits in each individual leaf (3), the minimum number of samples in each leaf (3).
XGBoost (144)	Number of the trees (8), maximum depth of each tree (3), a subsample of the training instances used in each iteration of boosting (6).
NGBoost (192)	Number of estimators (8), learning rate (4), a subsample of the training instances used in each iteration of boosting (6).
Artificial Neural Network (45)	Number of neurons per layer (5), number of hidden layers (3), learning rate (3).

Supplementary Table 1. Each classifier is fit with the K hyperparameter combinations shown above. The key hyperparameters for each model were identified and the number in brackets indicates how many values were tested for each hyperparameter.

Description of the sample-wise and feature-wise discrepancy measures

In this paper, we use nine statistics to measure the discrepancy between the imputed and true data. These fall into three classes; class A are sample-wise, class B are feature-wise and class C are derived from the sliced Wasserstein distances. For measuring all these discrepancies, after the imputation, both original and imputed data in the development and holdout sets are normalised to mean zero and unit standard deviation (SD) using the development set mean and SD.

Sample-wise metrics and their implementation. We briefly describe the sample-wise statistics used in the paper, along with giving details of the implementations used: **Root MSE (RMSE).** This is simply the square root of the mean square error, which is the average of the squared discrepancy errors between imputed and original samples. In this paper, we use the function `mean_squared_error` implemented in `sklearn` and take the square root of it. **Mean absolute error (MAE).** This is the average absolute difference between the imputed and the original values. In this paper, we use the function `mean_absolute_error` implemented in `sklearn`. **R^2.** This is the coefficient of determination, implemented in `sklearn` as `r2_score`, which measures the proportion of the variation in the imputed values that is predictable from the original values. This is expressed as a percentage. Note that this is not a metric.
Feature-wise metrics and their implementation. We briefly describe the distribution comparison measures used in the paper, along with giving details of the implementations used: **Kullback-Leibler (KL).** The KL divergence measures how different two probability distributions are from one another. This is implemented in Python using the standard calculation shown in `kl.py` in our codebase. **Kolmogorov-Smirnov (KS).** The KS test is used to assess whether two one-dimensional probability distributions differ. We use the function `ks_2samp` in the Python package `scipy.stats`. **Two-Wasserstein (2W).** The 2W distance[33] also measures the distance between two probability distributions using optimal transport. We use the function `emd2_1d` in the `POT` package in Python.

An example for calculation of sliced Wasserstein distances

We present here a small example to show how the Wasserstein distance calculations work. Here is a dataset with $N = 6$ samples and $d = 4$ features, one sample per row. The tables show the original dataset and the result of introducing missingness and

performing imputation. For example, we see from the tables that $\mathbf{x}_2 = (8,3,2,0)$ and $\hat{\mathbf{x}}_2 = (6,3,2,0)$.

i	$x^{(1)}$	$x^{(2)}$	$x^{(3)}$	$x^{(4)}$
1	3	2	5	1
2	8	3	2	0
3	5	5	3	2
4	1	9	4	1
5	3	0	3	3
6	4	5	6	3

Original data

i	$\hat{x}^{(1)}$	$\hat{x}^{(2)}$	$\hat{x}^{(3)}$	$\hat{x}^{(4)}$
1	3	1	6	1
2	6	3	2	0
3	5	5	3	2
4	1	7	4	2
5	6	4	3	3
6	4	5	6	2

Imputed data (with differences highlighted)

We now step through the calculations performed when running the following code on this data. (The data can be found in the `tests` directory of the Git repository.)

```
scripts/calculate_wasserstein.py --seed 20 --splits 4
  --directions 3 --output test-distances tests/original-data.csv
  tests/imputed-data.csv
```

The first step of the algorithm is to choose M random directions. We let $M = 3$ and the resulting directions are (rounded to 3 decimal places):

$$\mathbf{n}_1 = (-0.189, 0.632, 0.733, 0.167)$$
$$\mathbf{n}_2 = (0.289, -0.341, -0.637, -0.628)$$
$$\mathbf{n}_3 = (-0.728, 0.678, -0.041, 0.094)$$

We also choose P random partitions of $\{1,2,3,4,5,6\}$. Letting $P = 4$ gives the following partitions:

$$I_1 = \{1,3,4\} \qquad J_1 = \{2,5,6\}$$
$$I_2 = \{3,4,6\} \qquad J_2 = \{1,2,5\}$$
$$I_3 = \{2,3,6\} \qquad J_3 = \{1,4,5\}$$
$$I_4 = \{2,3,4\} \qquad J_4 = \{1,5,6\}$$

Note that I_1 and J_1 together include all of $\{1,2,3,4,5,6\}$ and similarly for the other pairs.

In step 2, we project all of the data onto the directions given by $\mathbf{n}_r$ for each r. Each projection is one-dimensional, so is a single number. For example, projecting $\mathbf{x}_2$ onto $\mathbf{n}_3$ gives

$$8 \times (-0.728) + 3 \times 0.678 + 2 \times (-0.041) + 0 \times 0.094 = -3.875$$

Performing this calculation for each sample and each direction gives the following pair of tables of $N \times M = 6 \times 3 = 18$ values each:

	direction		
i	$\mathbf{n}_1$	$\mathbf{n}_2$	$\mathbf{n}_3$
1	4.529	−3.631	−0.941
2	1.851	0.011	−3.875
3	4.747	−3.430	−0.188
4	8.596	−5.960	5.301
5	2.133	−2.929	−2.027
6	7.302	−6.258	0.510

Projected original data

	direction		
i	$\mathbf{n}_1$	$\mathbf{n}_2$	$\mathbf{n}_3$
1	4.631	−3.927	−1.66
2	2.229	−0.566	−2.418
3	4.747	−3.430	−0.188
4	7.499	−5.905	4.039
5	4.093	−3.428	−1.501
6	7.136	−5.631	0.417

Projected imputed data

Step 3 requires us to calculate the Wasserstein distances. We will do this for one example and then present a table of all the results. We need to pick a pair (r, p) with $r \in \{1, \ldots, M\}$ selecting the projection and $p \in \{1, \ldots, P\}$ selecting the partition. Let us choose $r = 1$ and $p = 4$.

The projection of the original data into the direction $\mathbf{n}_1$ is given by the first column of the above pair of tables and the partition is $I_4 = \{2,3,4\}$ and $J_4 = \{1,5,6\}$. The original data $\mathbf{x}_i.\mathbf{n}_r$ for $i \in I_p$ is therefore $\{1.851, 4.747, 8.596\}$. The standard

deviation of this set of data is 2.763, so for the following Wasserstein distance calculations, we first normalise the data by dividing by this. The original data for this direction thus becomes

$$\{1.640, 0.670, 1.718, 3.112, 0.772, 2.643\}$$

and the imputed data becomes

$$\{1.676, 0.807, 1.718, 2.714, 1.482, 2.583\}.$$

To find $w(1,4)$, we find the 2-Wasserstein distance between the I_4 terms and the J_4 ones of the scaled original data, that is between $\{0.670, 1.718, 3.112\}$ and $\{1.640, 0.772, 2.643\}$. Using the Python Optimal Transport (POT) library function `emd2_1d`, we find that this is 0.079. Similarly, to find $\hat{w}(1,4)$, we find the 2-Wasserstein distance between the I_4 terms of the scaled original data and the J_4 terms of the ones of the scaled imputed data, that is between $\{0.670, 1.718, 3.112\}$ and $\{1.676, 1.482, 2.583\}$, which is 0.313.

Doing this for all of the choices of r and p gives the following baseline distances and imputed distances:

p	direction (r) 1	2	3
1	1.498	3.142	1.505
2	6.733	5.785	3.303
3	0.121	0.445	2.426
4	0.079	0.488	0.632

Baseline Wasserstein distances $w(r,p)$

p	direction (r) 1	2	3
1	0.750	2.112	1.196
2	4.219	4.152	3.133
3	0.341	0.619	1.719
4	0.313	0.678	0.715

Imputed data Wasserstein distances $\hat{w}(r,p)$

Outlier Analysis Details

To isolate whether the large variances are due to stochasticity in the algorithms, we now go back and consider the original feature distributions, rather than the projected distributions. If an imputation algorithm occasionally imputes poorly in particular features, it will be identified here. For each holdout and validation set, we compute the Wasserstein distance between the imputed data and the true data for all features in the 10 repeats, i.e. for each feature we have 10 Wasserstein distances. We want to understand how often these distances are very large relative to how often they are small. In Figure 8, we show the proportion of the imputations that have Wasserstein distances above a threshold of 10^{-7} for each holdout set and validation set. The plots for other thresholds are in Supplementary Figure 3. It can be seen that the mean imputation method leads to feature imputations that always have relatively large distances from the true values. This is not surprising as it is the baseline model. It is surprising that GAIN is often imputing with high distance (80% at 10^{-7} and 40% at 10^{-6}), indicating some stochasticity in the imputation method which causes poor imputations for some computations and better imputation for others. MIWAE demonstrates the same stochasticity to a lesser extent, followed by MissForest. This highlights the importance of performing multiple imputation runs for models which have stochasticity integral to them. For GAIN and MIWAE, this is particularly true as deep neural networks will occasionally find local minima at their optimum and generative adversarial networks are liable to mode collapse[34].

Interpretability

First, for each classifier, we find the top ten configurations of the validation set, holdout set and imputation methods that achieve the best performance. We then rank these configurations by the distance ratio induced by the imputation method and take the model with the smallest and largest induced distance ratio for the sliced Wasserstein distance. This gives us the two models which are high performing but which are trained using data of different imputation quality (see Tables 7–9). The features that are important for a model's prediction can be found using many interpretability techniques. In this paper, we employ Shapley values[35] implemented in the Python package `shap`.

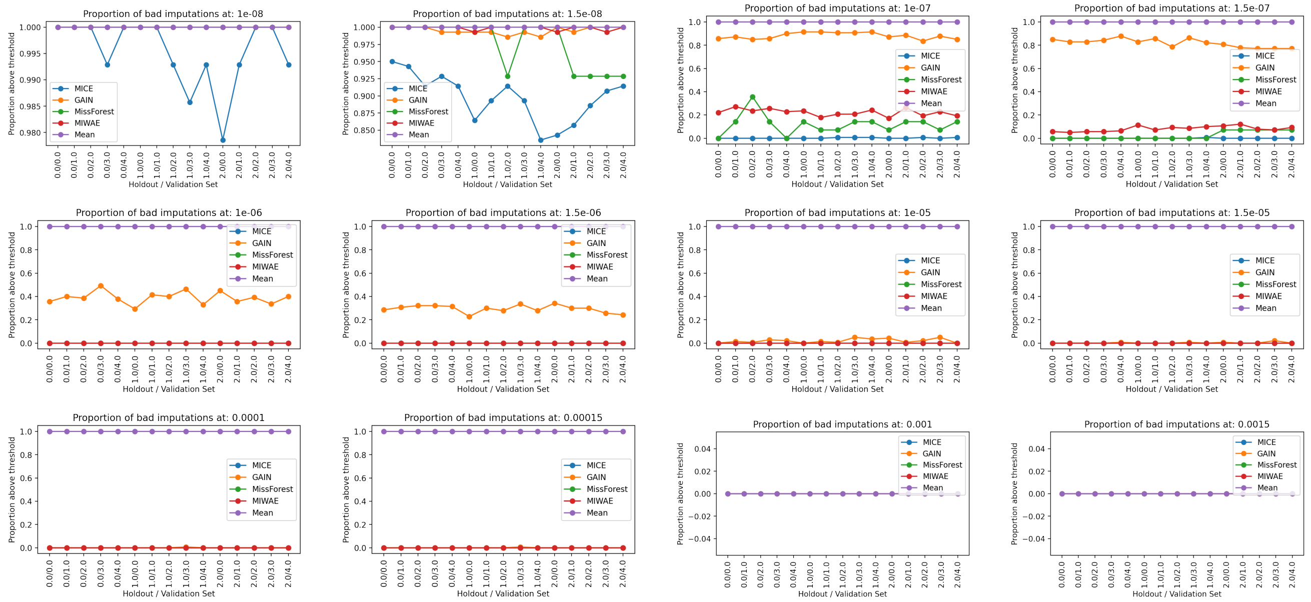

Supplementary Figure 3. The proportion of repeated imputations that give outlier Wasserstein distances, at the thresholds shown, for different imputation methods.

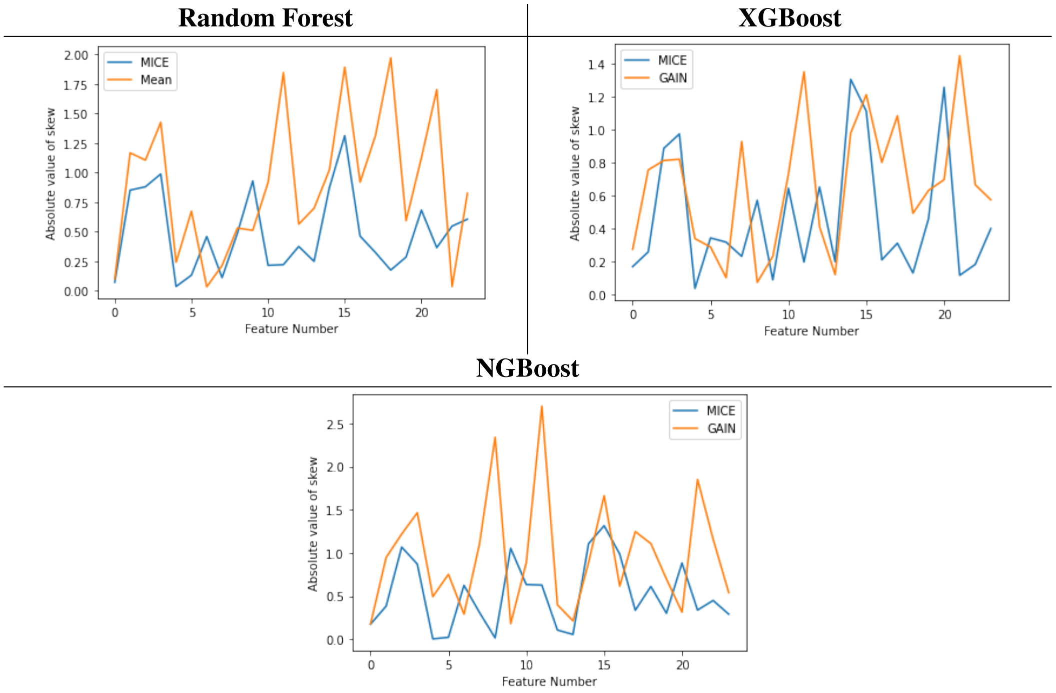

Supplementary Figure 4. For each classifier, we give the absolute skew of the Shapley values for each feature for the two candidate models identified. In the `shap` Python package used for calculating these values, the `TreeExplainer` functionality is not implemented for the Neural Network and Logistic Regression classifiers.

Software version and imputation tools

All of these experiments are run within a Conda environment, and required Python packages can be installed by using the requirement.txt file shared in our repository (**available upon acceptance**). Additionally, we used Python[36] version 3.10.4, RStudio[37] version 4.2 (with the MICE R library[38] version 3.14.0) and corrected implementations of GAIN and MIWAE which can be found in our repository. The implementation of MissForest included in `missingpy` version 0.2.0 is adopted and the

mean imputation is implemented by using the `scikit-learn` package[4] version 1.1.1. For the classification methods, all classifiers RandomForest, Logistic Regression, and Neural Network are implemented by using the `scikit-learn` package[4] version 1.1.1. For XGBoost and NGBoost, versions 1.6.1 and 0.3.12 are used respectively.

Supplementary Figures

Additional performance metrics for the downstream classification task

In addition to exploring the AUC values for the downstream performance of classifiers, we also report the **Accuracy**, **Brier Score**, **Precision**, **Sensitivity** and **Specificity**. These results are presented in Supplementary Figures 5–9.

Dependence of the accuracy

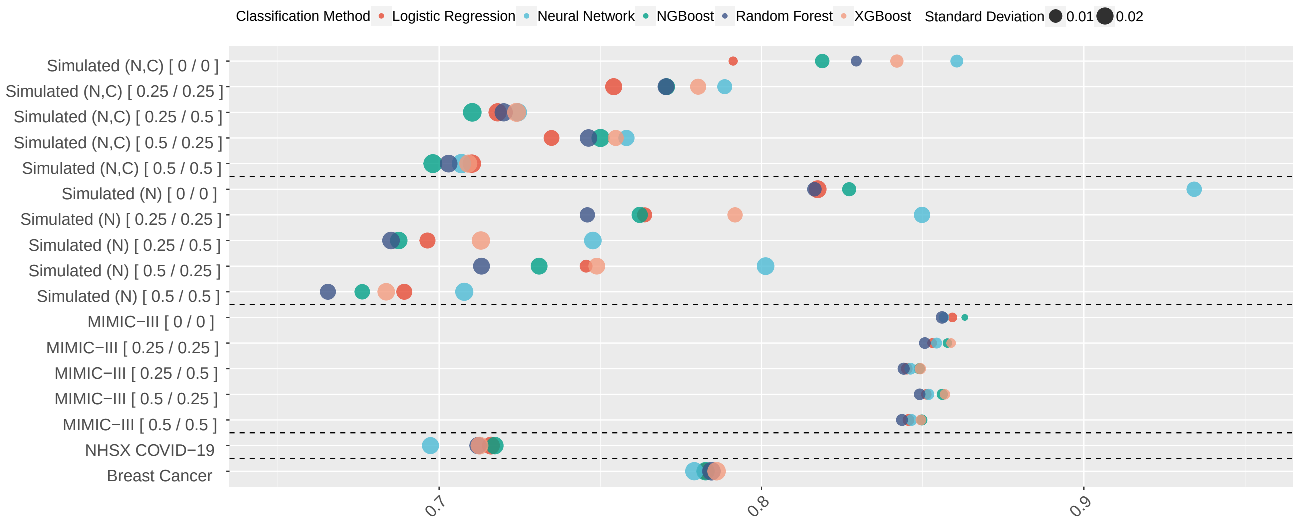

(a) Classifier dependence

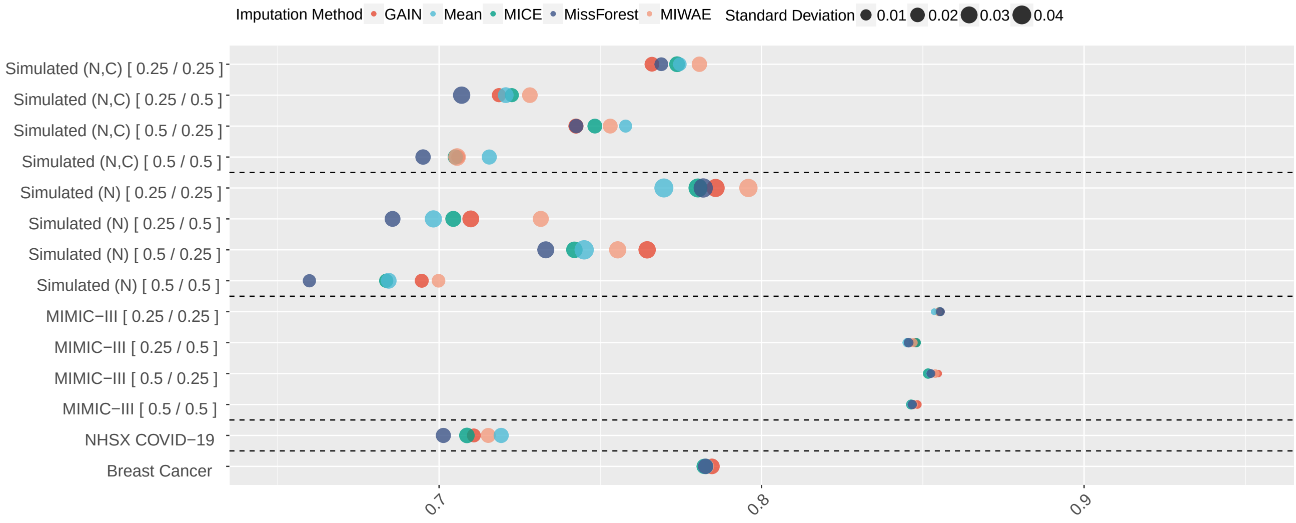

(b) Imputation dependence

Supplementary Figure 5. Dependence of the classification accuracy on the (a) classification and (b) imputation methods. The size of each marker indicates the standard deviation.

Dependence of the Brier score

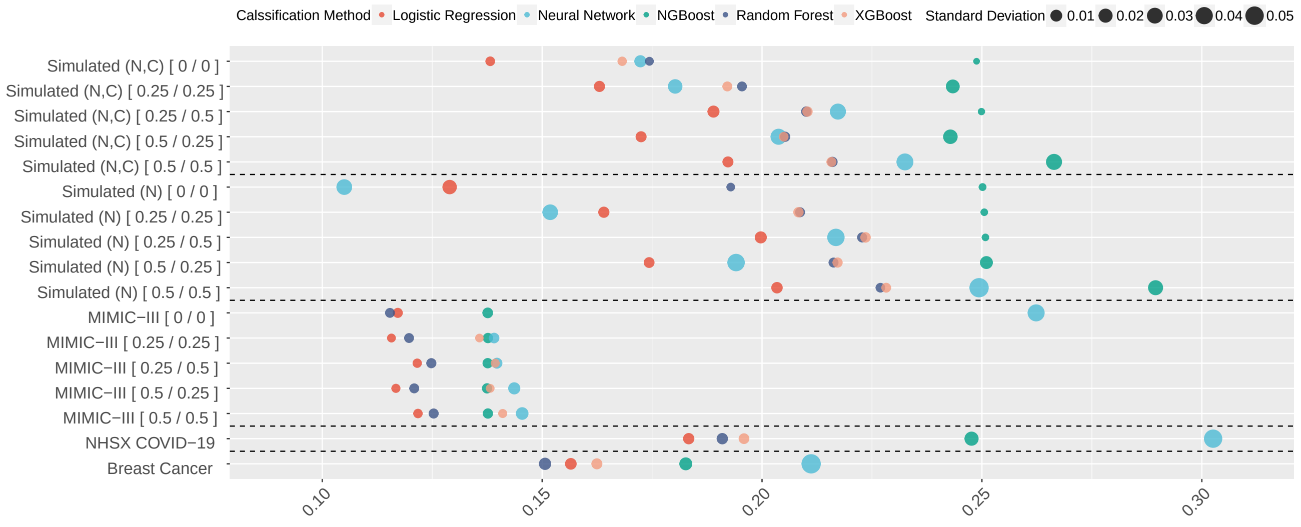

(a) Classifier dependence

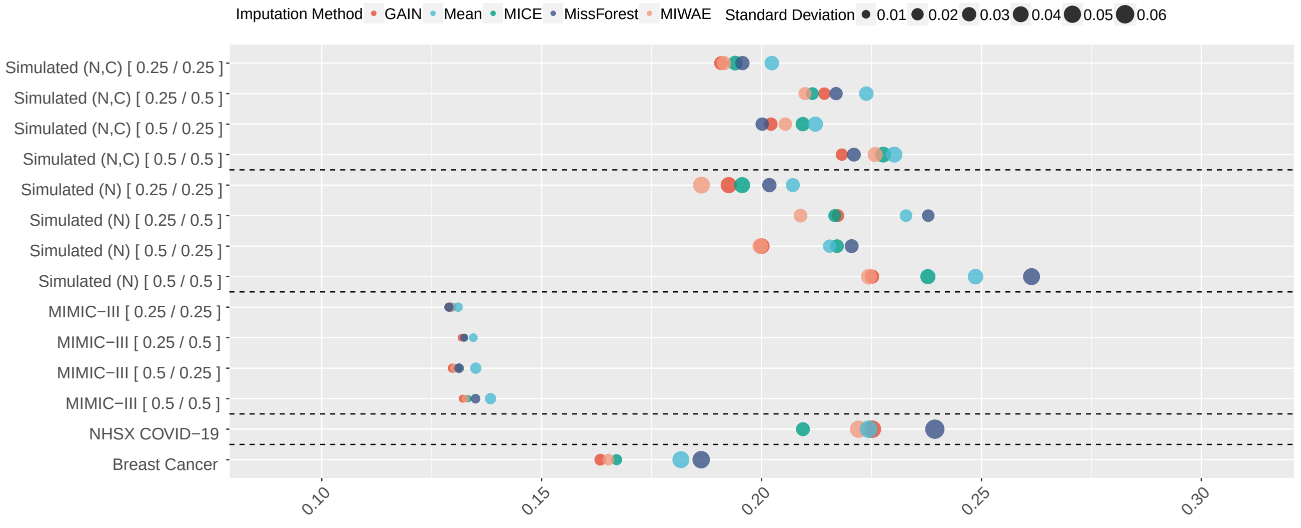

(b) Imputation dependence

Supplementary Figure 6. Dependence of the classification Brier score on the (a) classification and (b) imputation methods. The size of each marker indicates the standard deviation.

Dependence of the precision

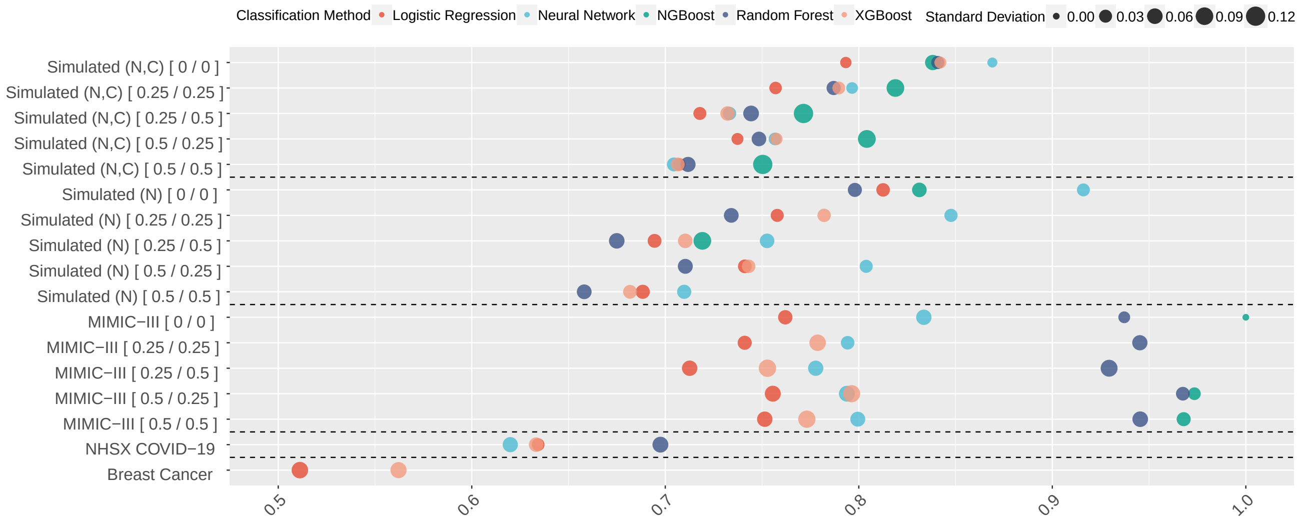

(a) Classifier dependence

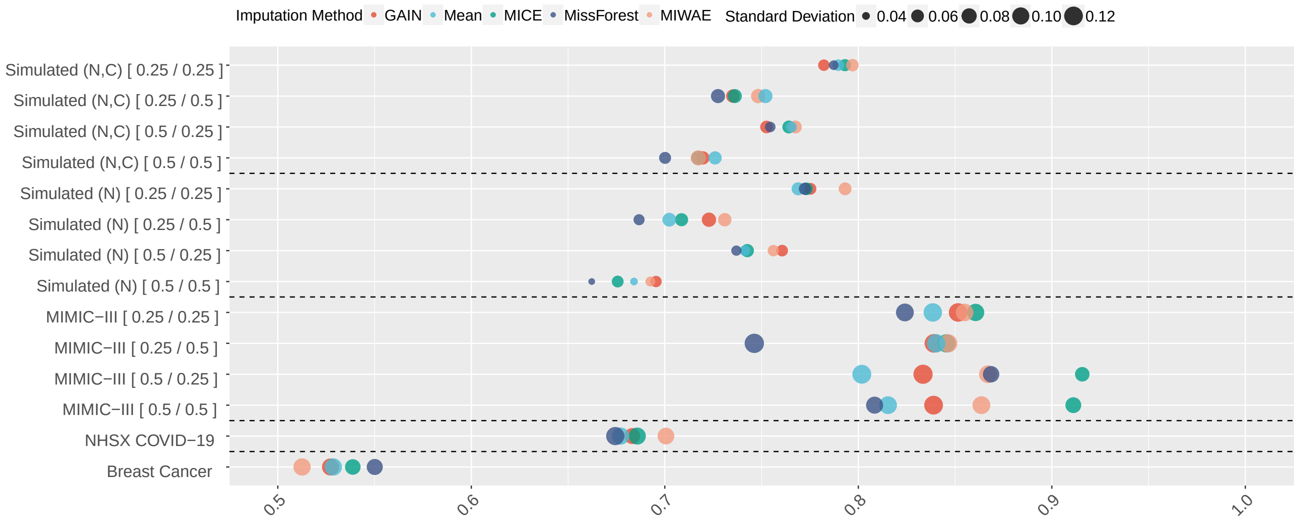

(b) Imputation dependence

Supplementary Figure 7. Dependence of the classification precision on the (a) classification and (b) imputation methods. The size of each marker indicates the standard deviation.

Dependence of the sensitvity

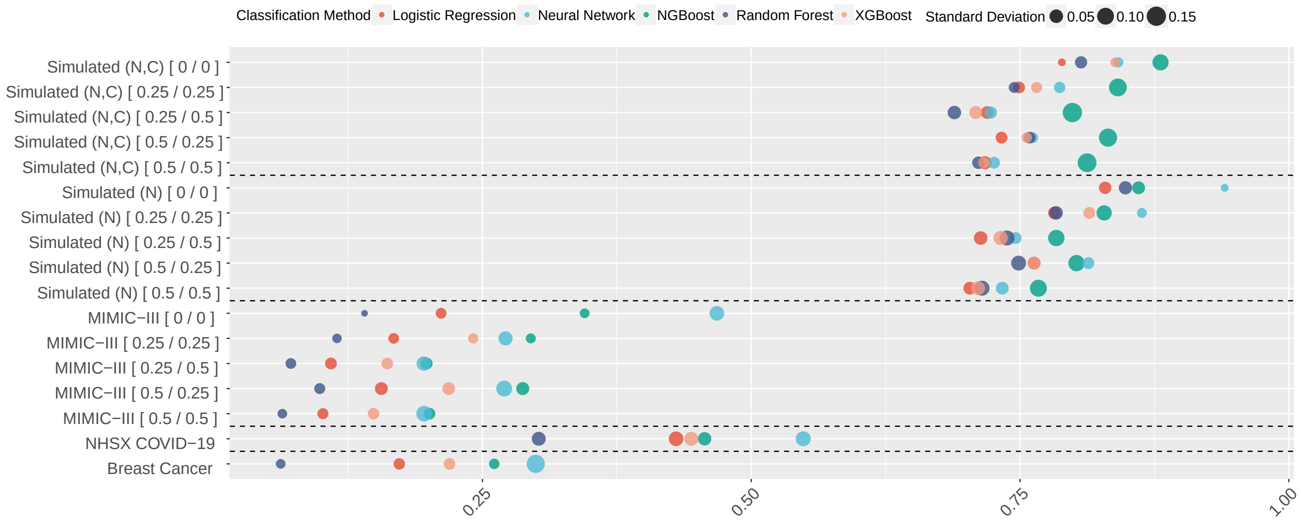

(a) Classifier dependence

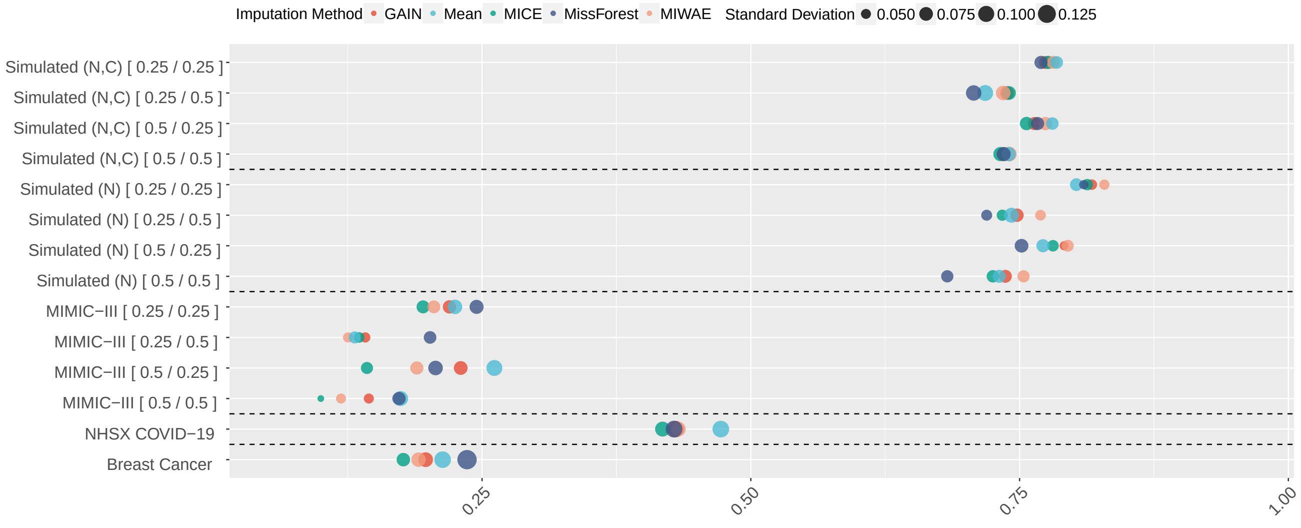

(b) Imputation dependence

Supplementary Figure 8. Dependence of the classification sensitivity on the (a) classification and (b) imputation methods. The size of each marker indicates the standard deviation.

Dependence of the specificity

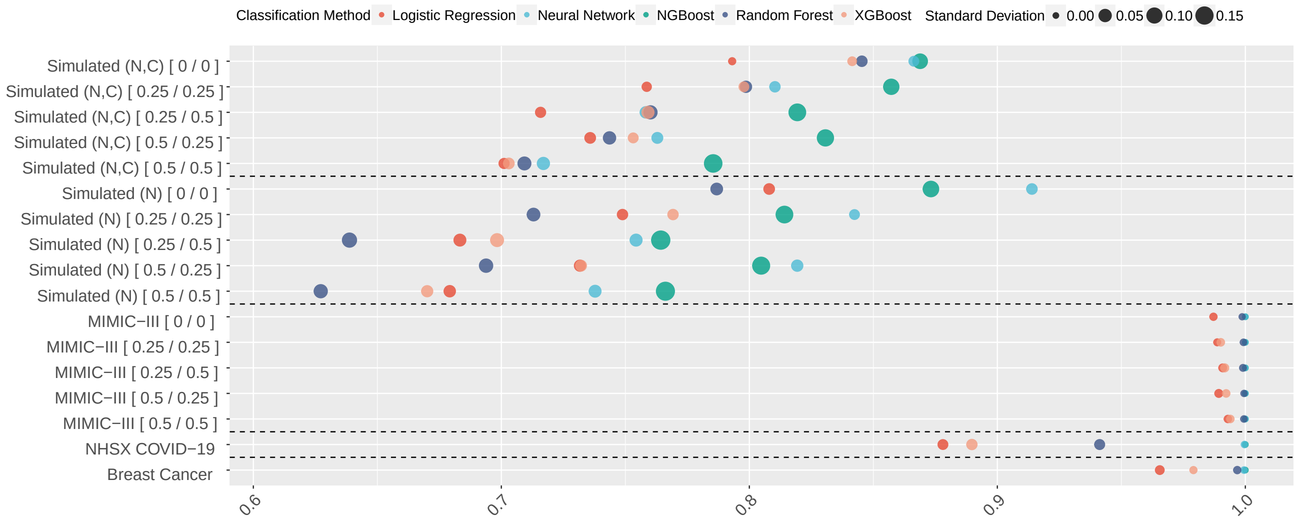

(a) Classifier dependence

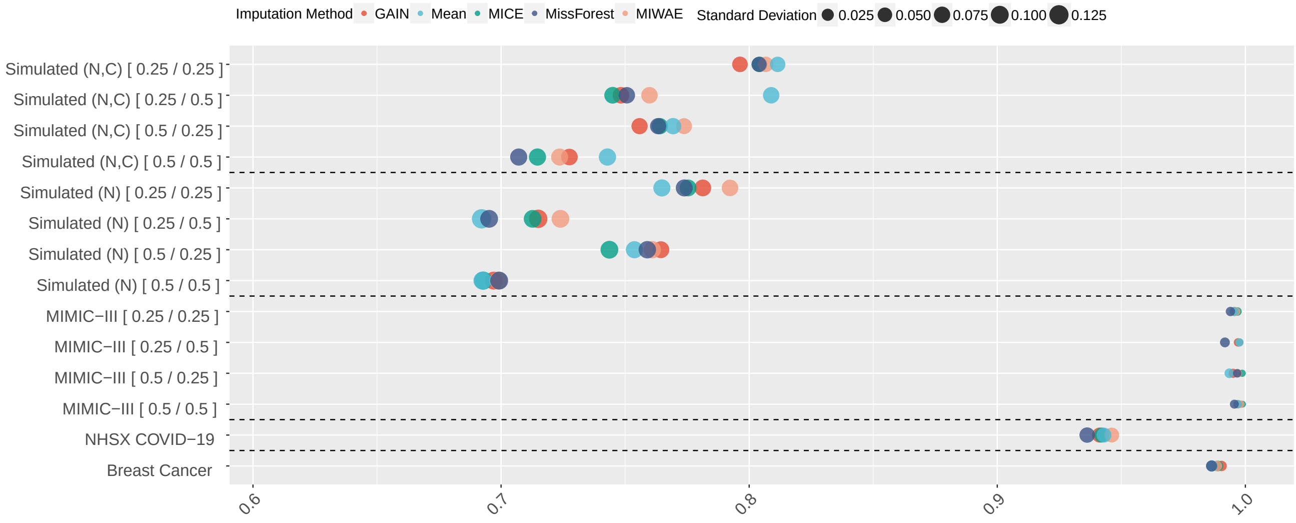

(b) Imputation dependence

Supplementary Figure 9. Dependence of the classification specificity on the (a) classification and (b) imputation methods. The size of each marker indicates the standard deviation.

Sample-wise discrepancy for the MIMIC-III dataset at different train and test missingness rates

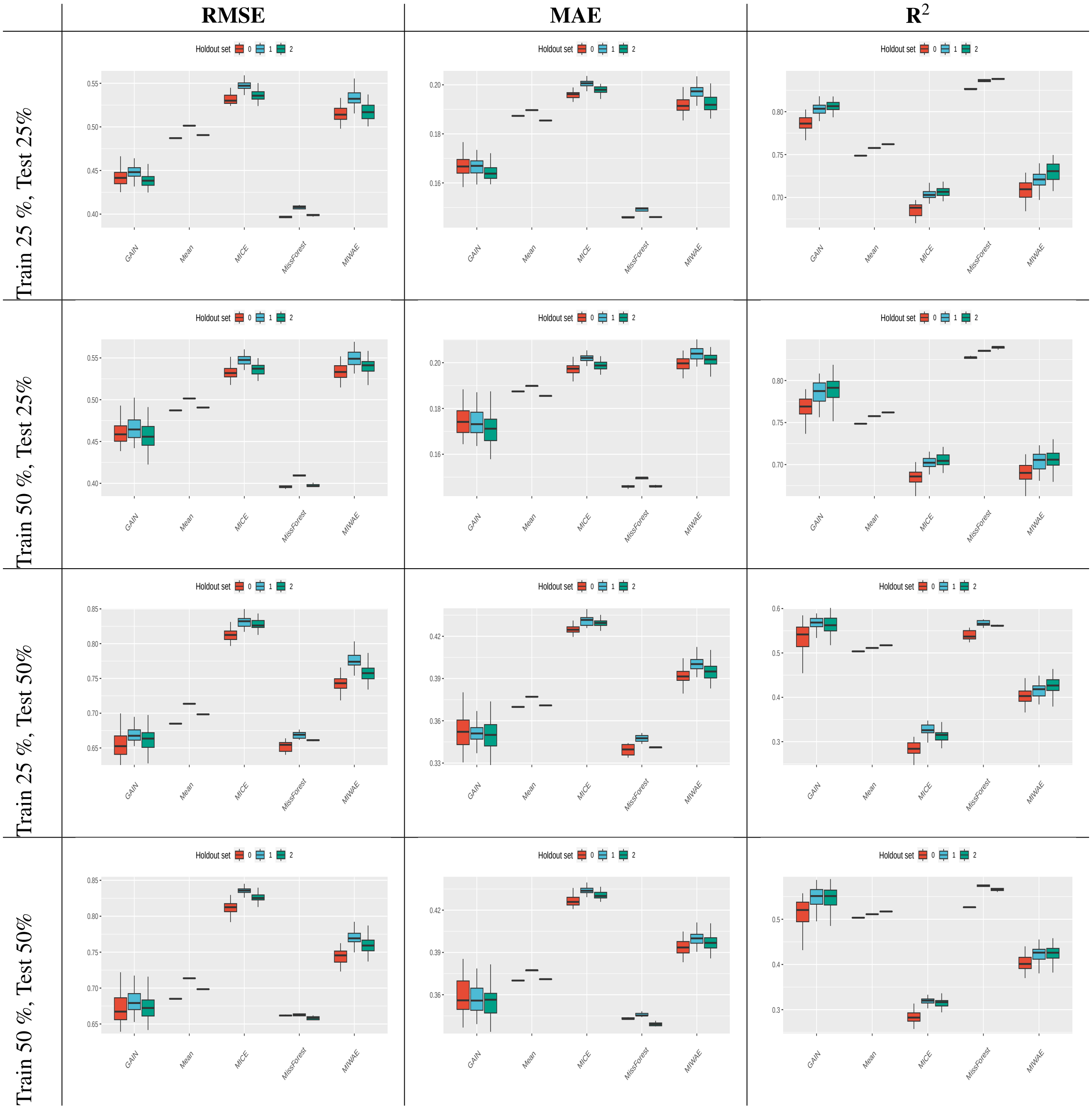

Supplementary Figure 10. The sample-wise statistics for the **MIMIC-III** dataset at the different train and test missingness rates considered. Whiskers extend to the extreme values, with outliers omitted that are above 1.5 times the interquartile range from the median (horizontal line).

Sample-wise discrepancy for the Simulated (N) dataset at different train and test missingness rates

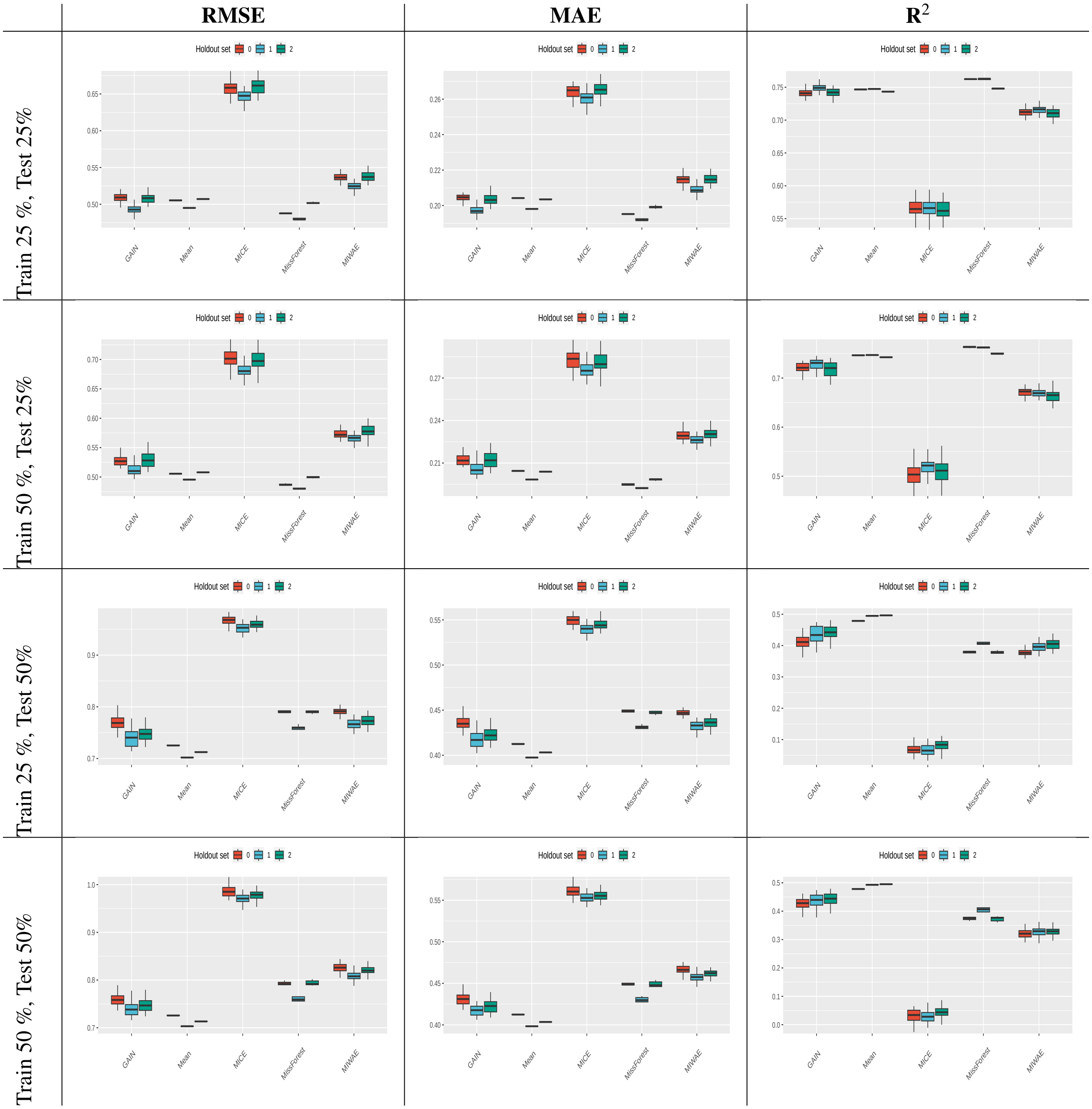

Supplementary Figure 11. The sample-wise statistics for the **Simulated (N)** dataset at the different train and test missingness rates considered. Whiskers extend to the extreme values, with outliers omitted that are above 1.5 times the interquartile range from the median (horizontal line).

Sample-wise discrepancy for the Simulated (N,C) dataset at different train and test missingness rates

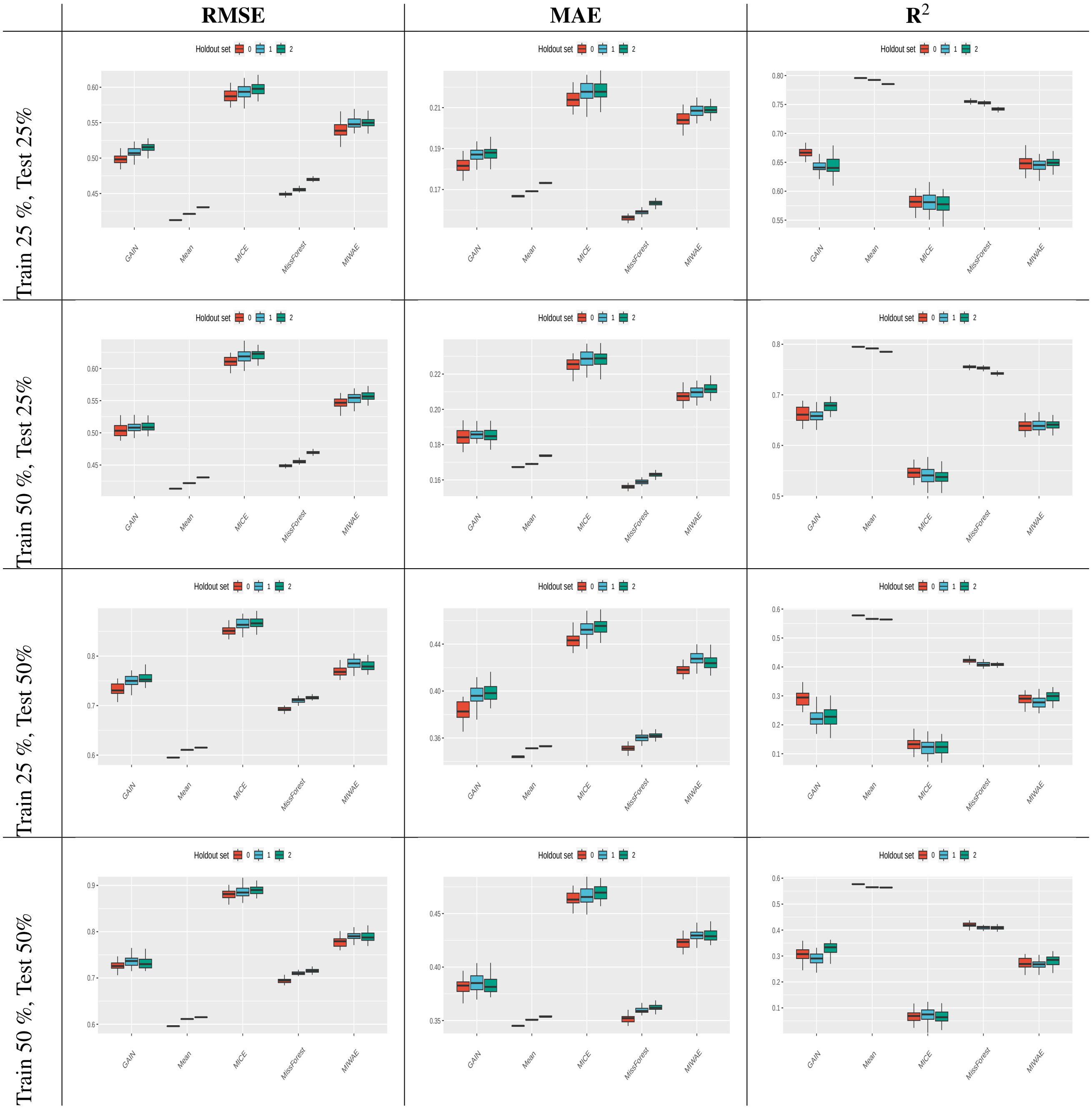

Supplementary Figure 12. The sample-wise statistics for the **Simulated (N,C)** dataset at the different train and test missingness rates considered. Whiskers extend to the extreme values, with outliers omitted that are above 1.5 times the interquartile range from the median (horizontal line).

Feature-wise discrepancy for the MIMIC-III dataset at the respective train and test missingness rates of 25% and 50%.

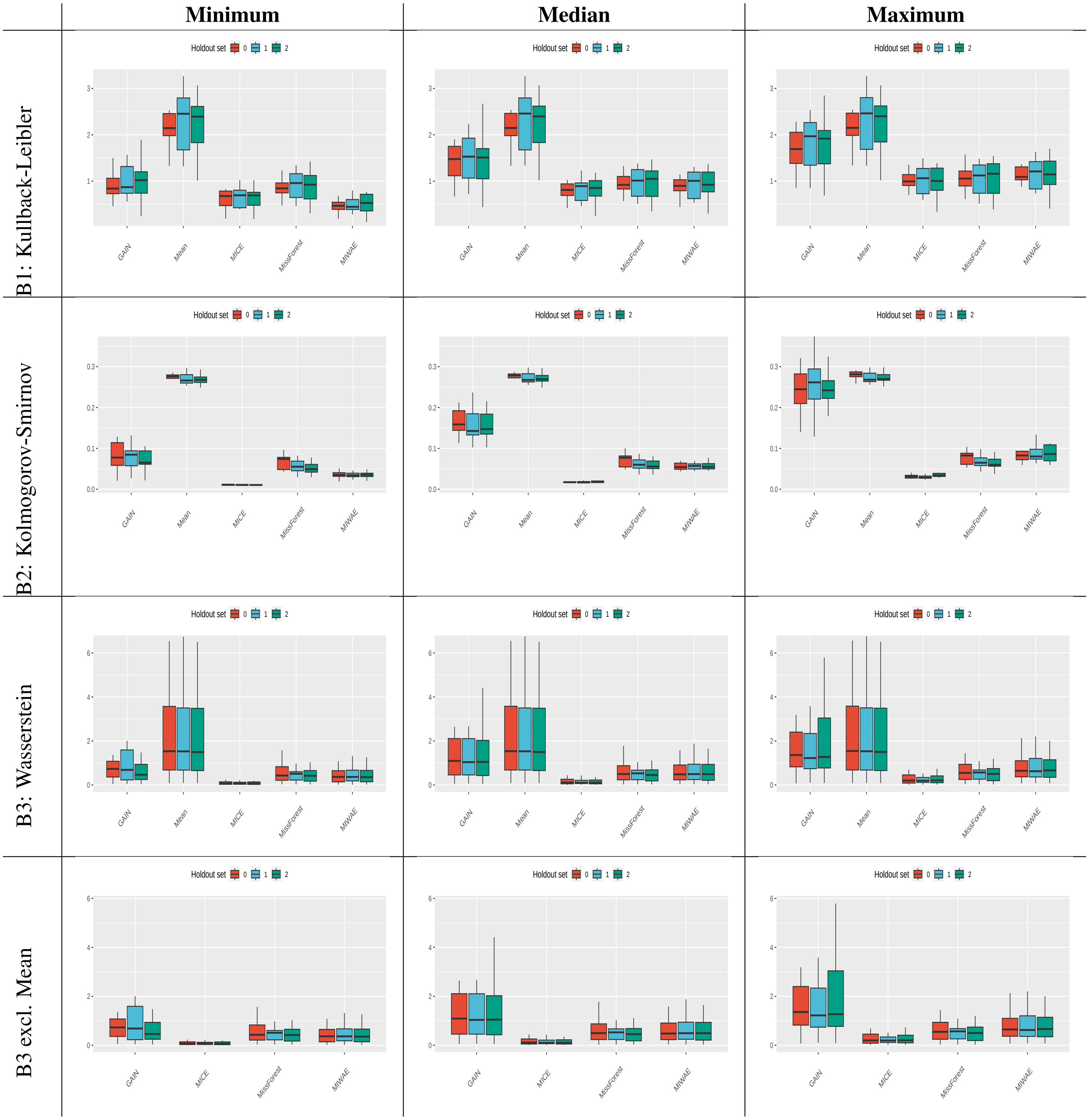

Supplementary Figure 13. Feature-wise 25% train missingness and 50% test missingness. Whiskers extend to the extreme values, with outliers omitted that are above 1.5 times the interquartile range from the median (horizontal line).

Feature-wise discrepancy for the MIMIC-III dataset at the respective train and test missingness rates of 50% and 25%.

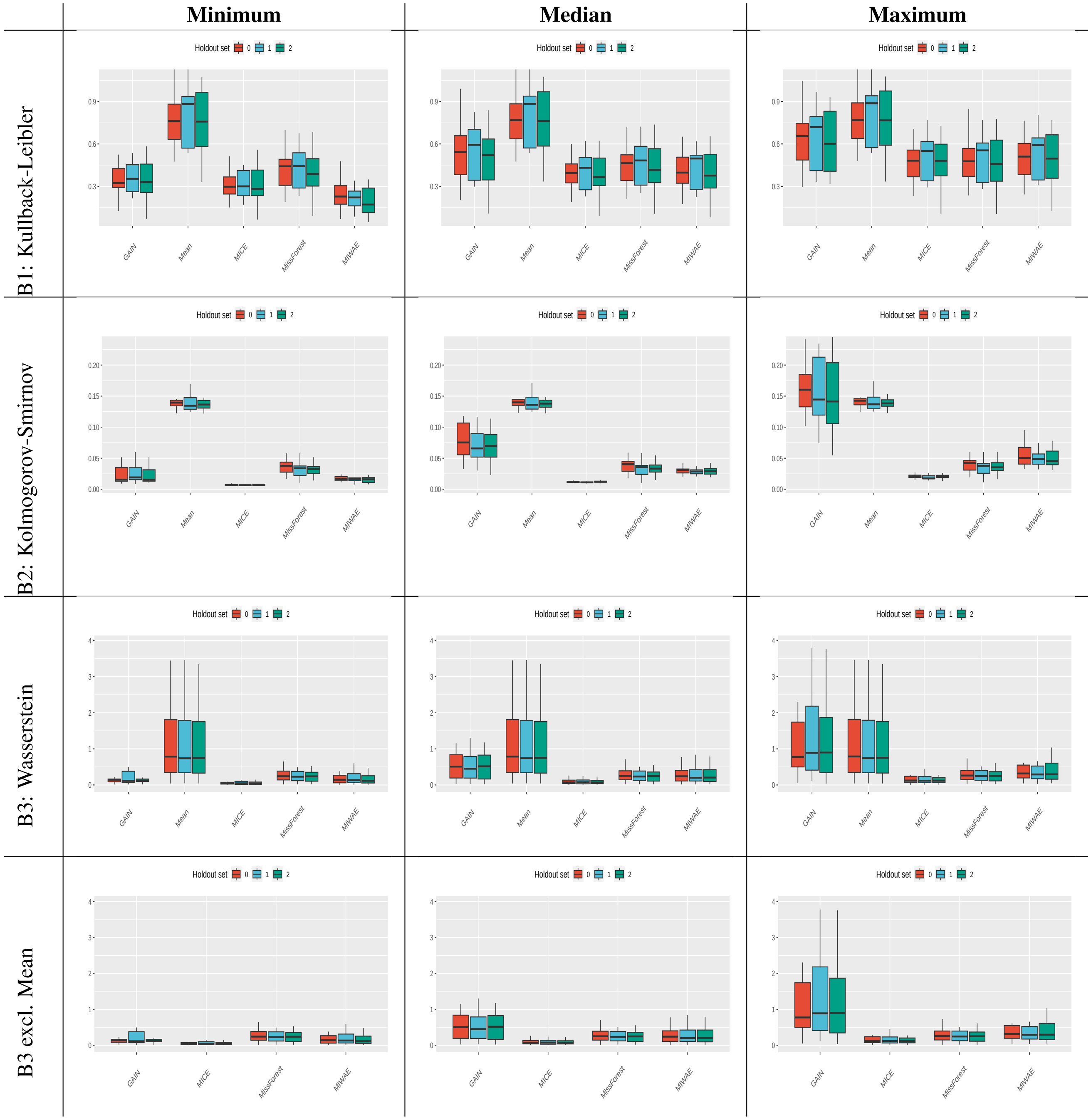

Supplementary Figure 14. Feature-wise 50% train missingness and 25% test missingness. Whiskers extend to the extreme values, with outliers omitted that are above 1.5 times the interquartile range from the median (horizontal line).

Feature-wise discrepancy for the MIMIC-III dataset at the respective train and test missingness rates of 50% and 50%.

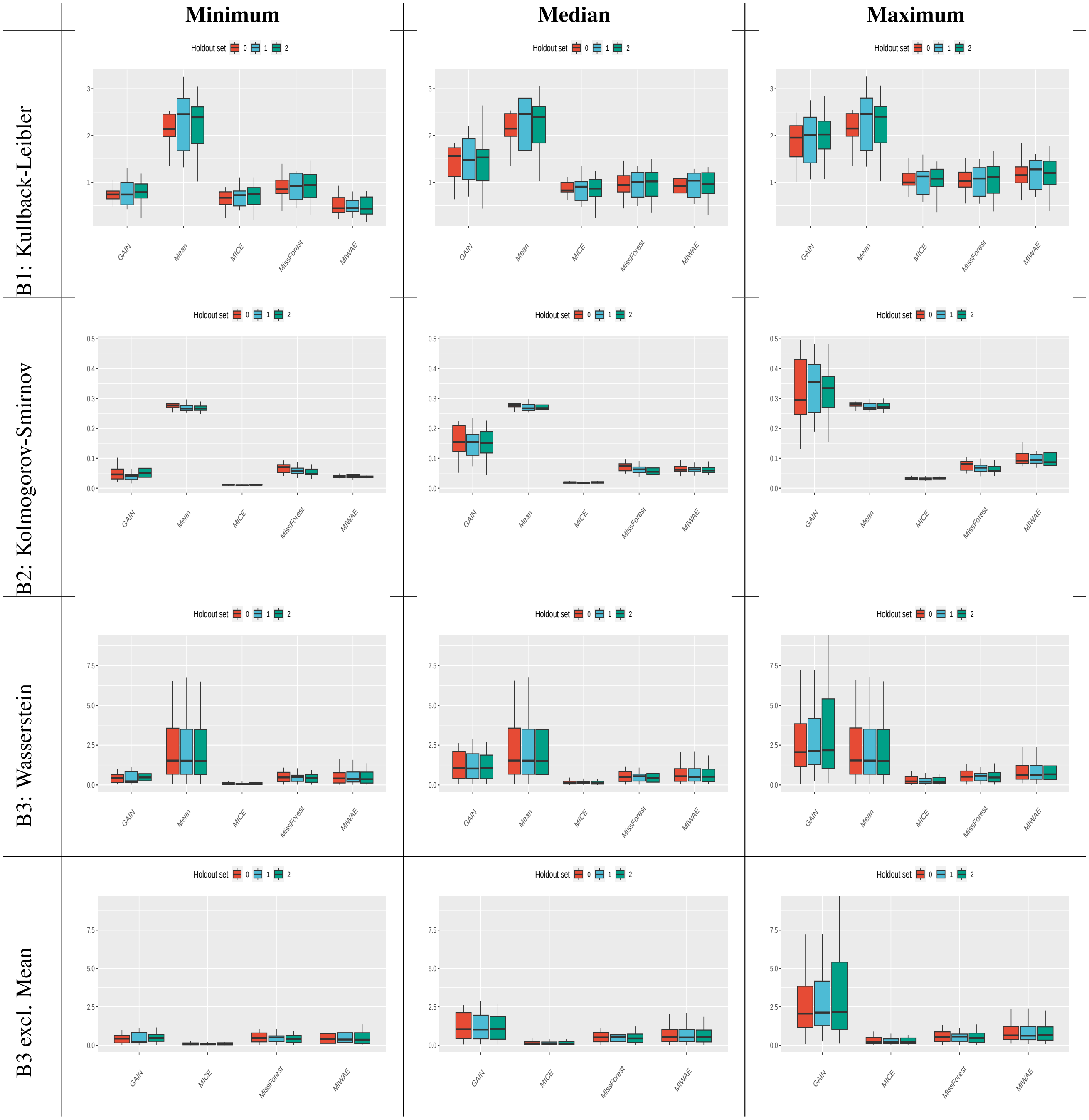

Supplementary Figure 15. Feature-wise 50% train missingness and 50% test missingness. Whiskers extend to the extreme values, with outliers omitted that are above 1.5 times the interquartile range from the median (horizontal line).

Feature-wise discrepancy for the Simulated (N) at the respective train and test missingness rates of 25% and 25%.

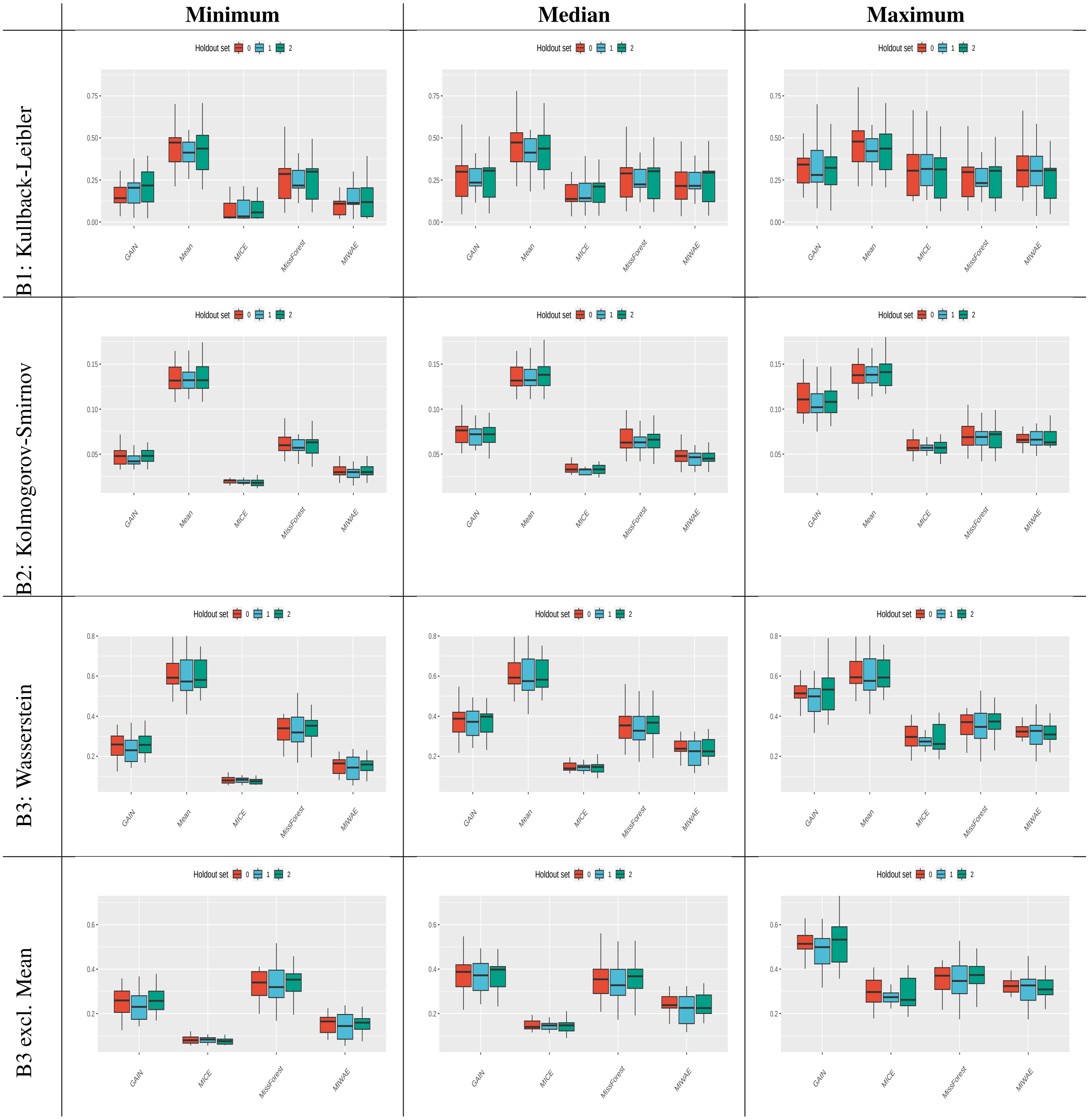

Supplementary Figure 16. Feature-wise 25% train missingness and 25% test missingness. Whiskers extend to the extreme values, with outliers omitted that are above 1.5 times the interquartile range from the median (horizontal line).

Feature-wise discrepancy for the Simulated (N) dataset at the respective train and test missingness rates of 25% and 50%.

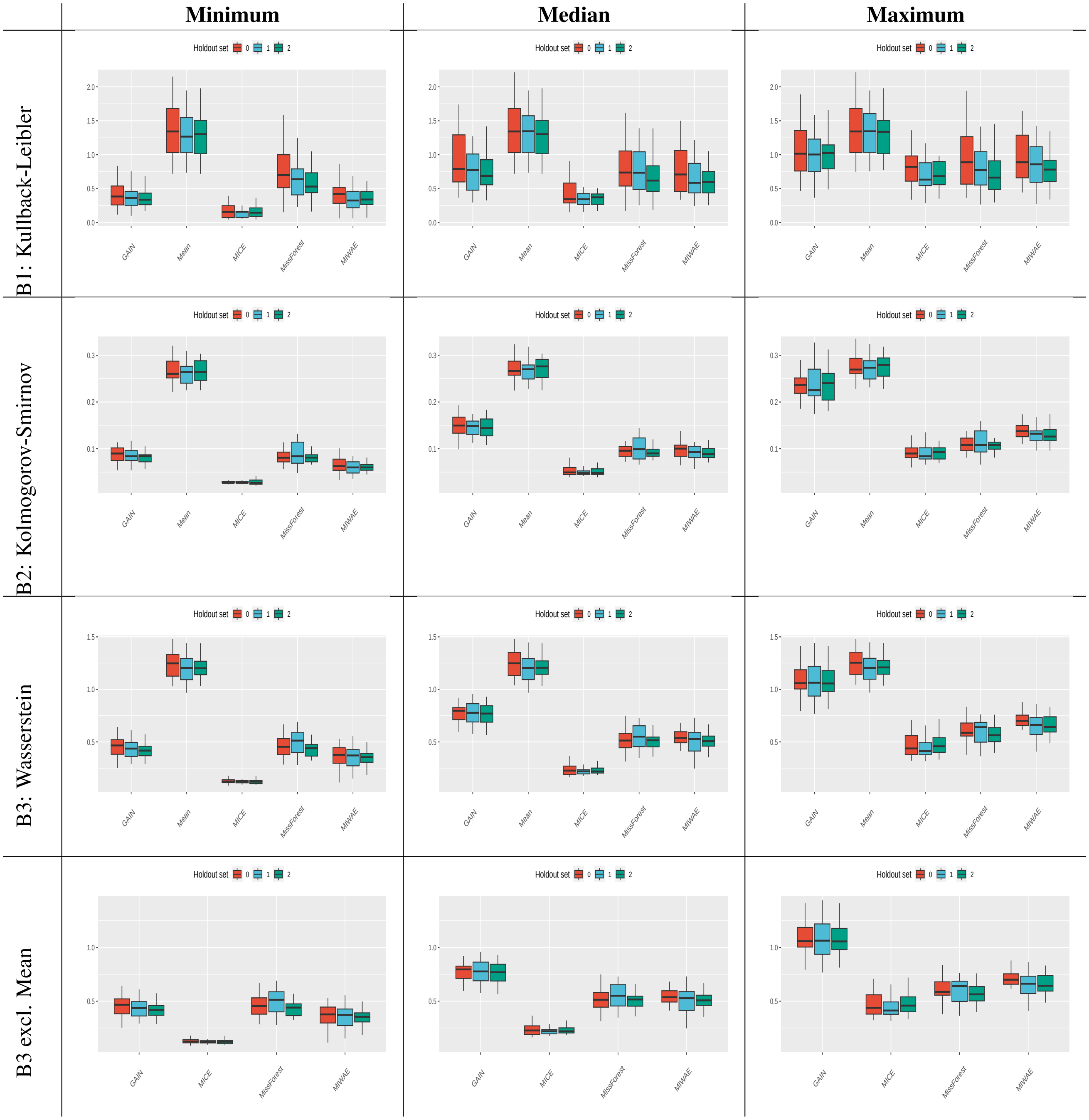

Supplementary Figure 17. Feature-wise 25% train missingness and 50% test missingness. Whiskers extend to the extreme values, with outliers omitted that are above 1.5 times the interquartile range from the median (horizontal line).

Feature-wise discrepancy for the Simulated (N) dataset at the respective train and test missingness rates of 50% and 25%.

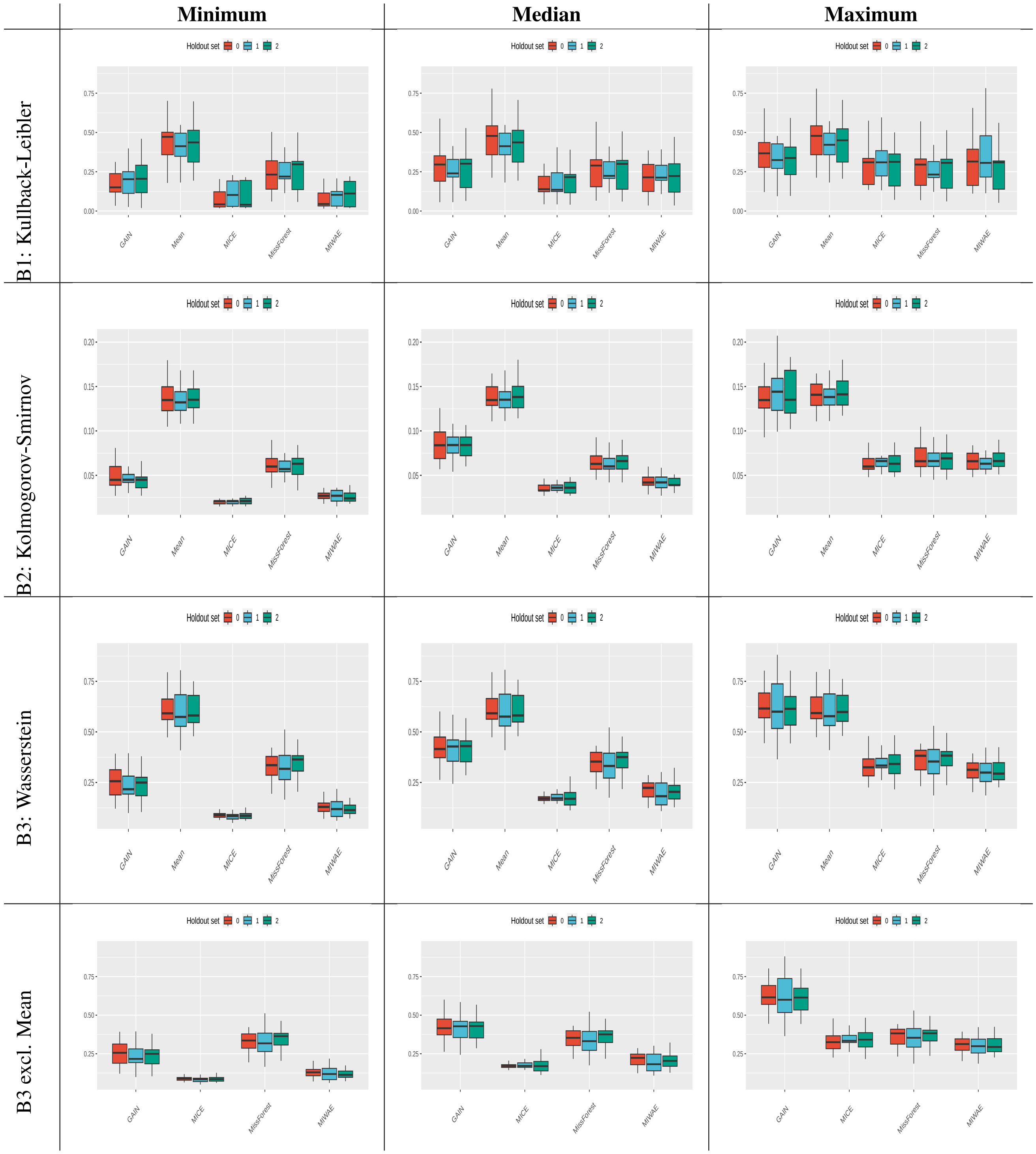

Supplementary Figure 18. Feature-wise 50% train missingness and 25% test missingness. Whiskers extend to the extreme values, with outliers omitted that are above 1.5 times the interquartile range from the median (horizontal line).

Feature-wise discrepancy for the Simulated (N) dataset at the respective train and test missingness rates of 50% and 50%.

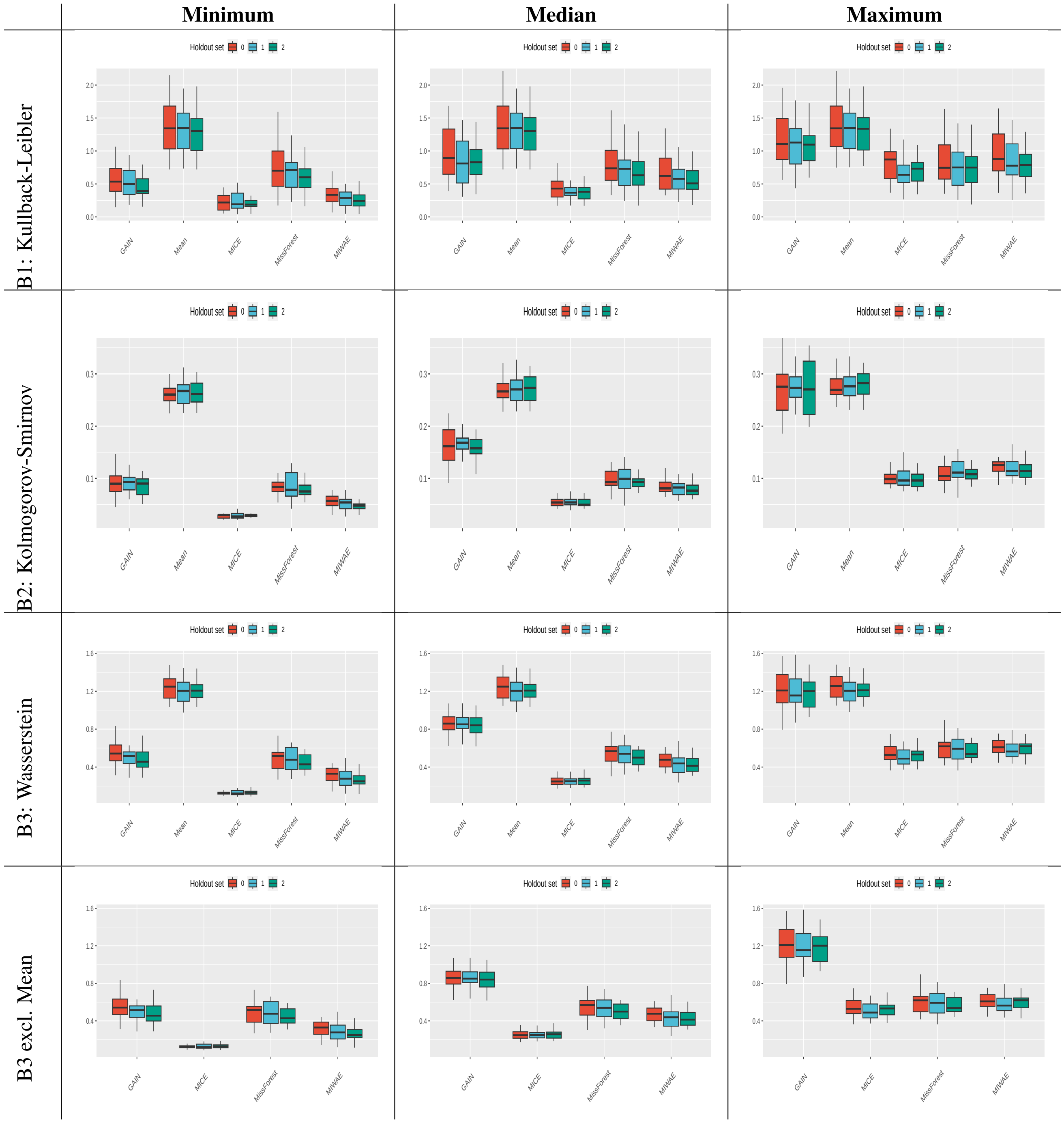

Supplementary Figure 19. Feature-wise 50% train missingness and 50% test missingness. Whiskers extend to the extreme values, with outliers omitted that are above 1.5 times the interquartile range from the median (horizontal line).

Feature-wise discrepancy for the Simulated (N,C) dataset at the respective train and test missingness rates of 25% and 25%.

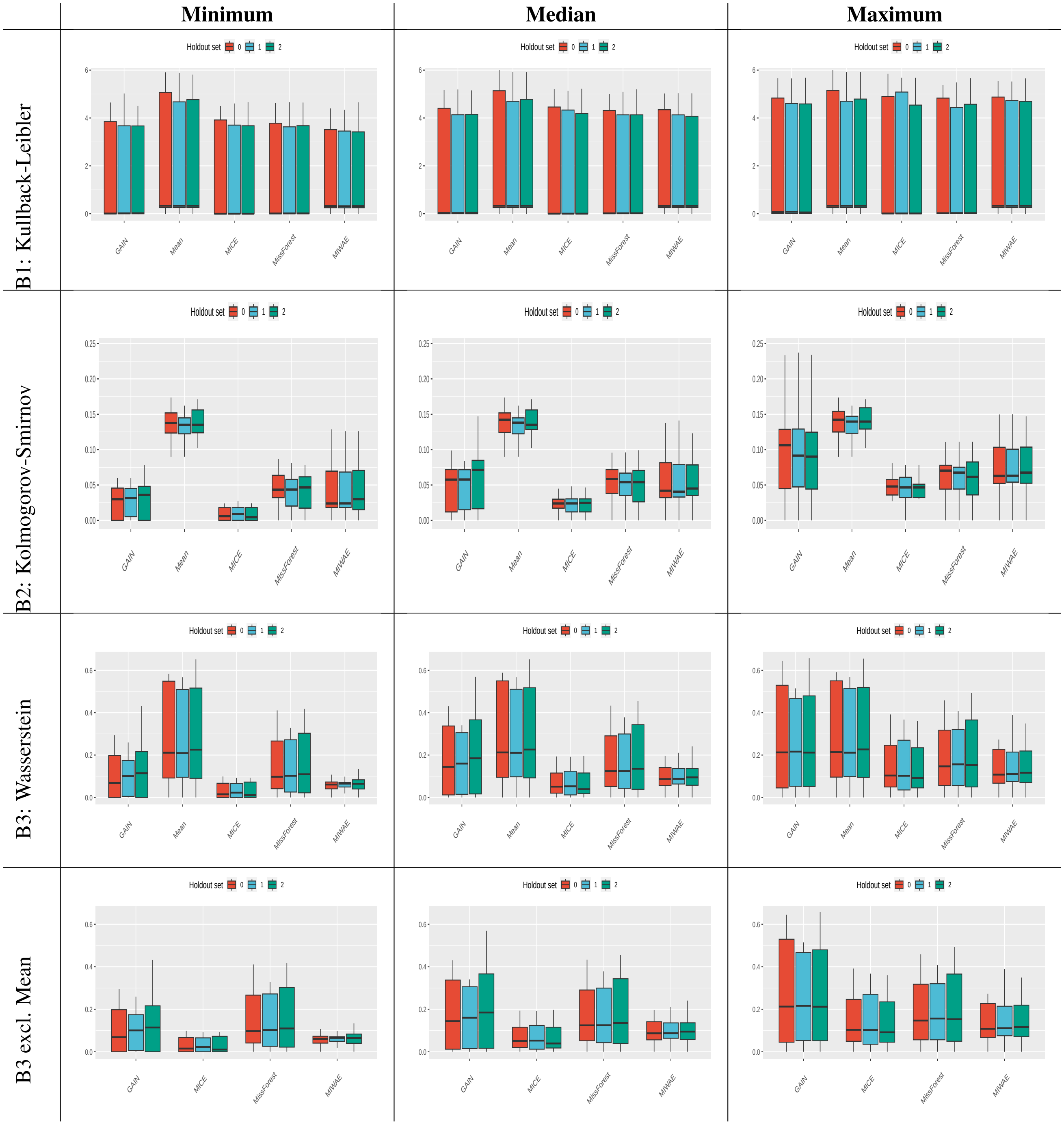

Supplementary Figure 20. Feature-wise 25% train missingness and 25% test missingness. Whiskers extend to the extreme values, with outliers omitted that are above 1.5 times the interquartile range from the median (horizontal line).

Feature-wise discrepancy for the Simulated (N,C) dataset at the respective train and test missingness rates of 25% and 50%.

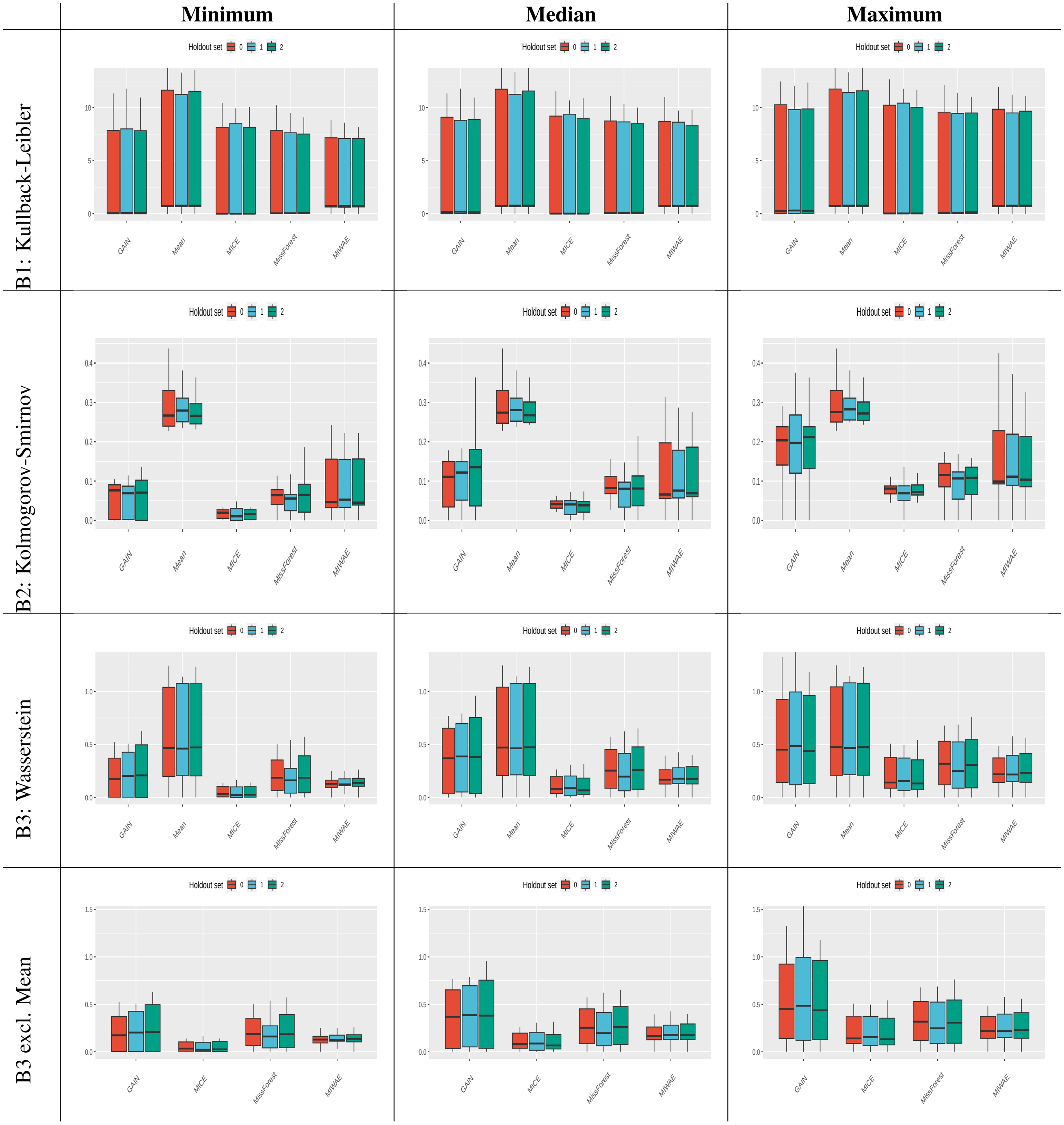

Supplementary Figure 21. Feature-wise 25% train missingness and 50% test missingness. Whiskers extend to the extreme values, with outliers omitted that are above 1.5 times the interquartile range from the median (horizontal line).

Feature-wise discrepancy for the Simulated (N,C) dataset at the respective train and test missingness rates of 50% and 25%.

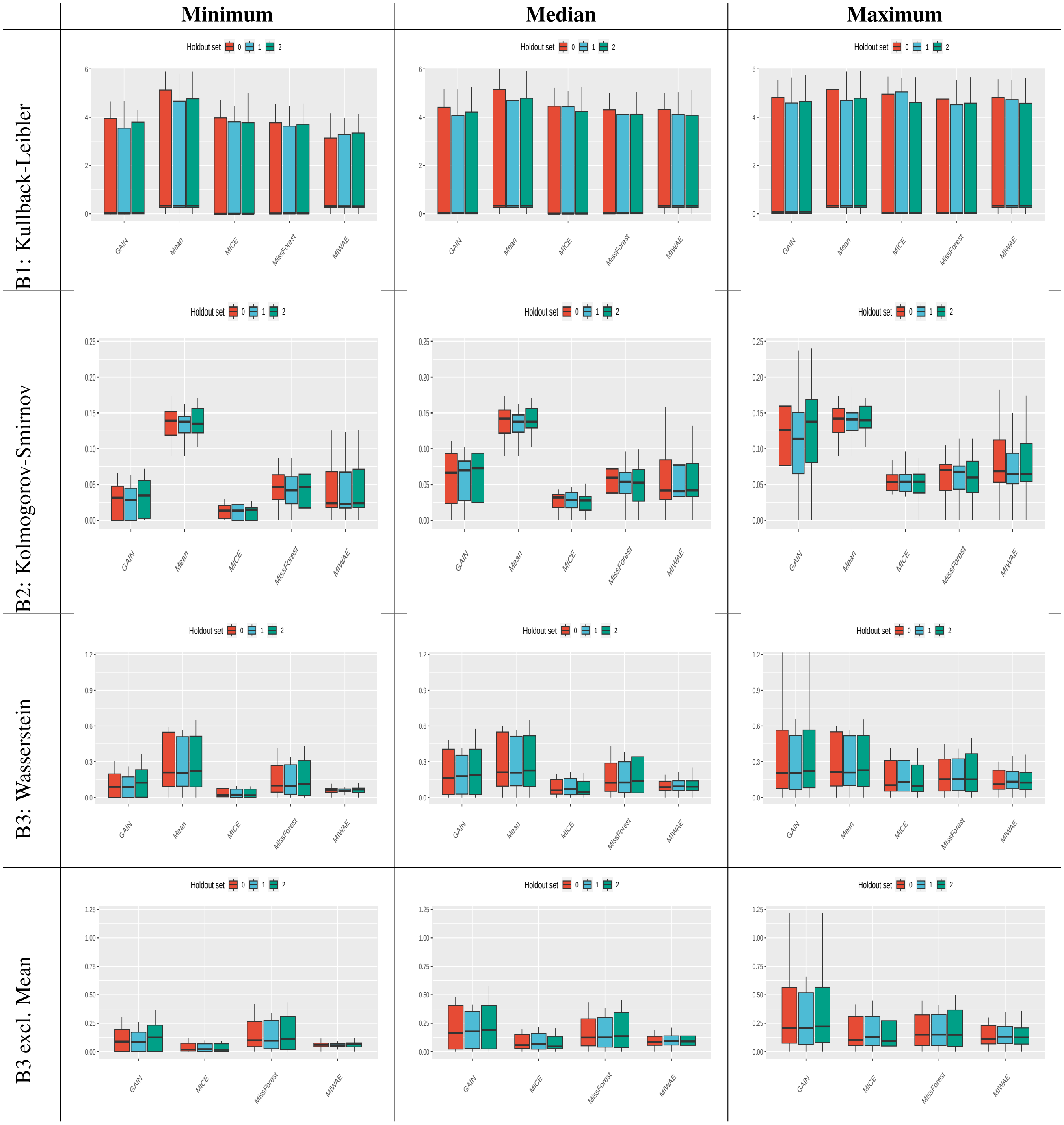

Supplementary Figure 22. Feature-wise 50% train missingness and 25% test missingness. Whiskers extend to the extreme values, with outliers omitted that are above 1.5 times the interquartile range from the median (horizontal line).

Feature-wise discrepancy for the Simulated (N,C) dataset at the respective train and test missingness rates of 50% and 50%.

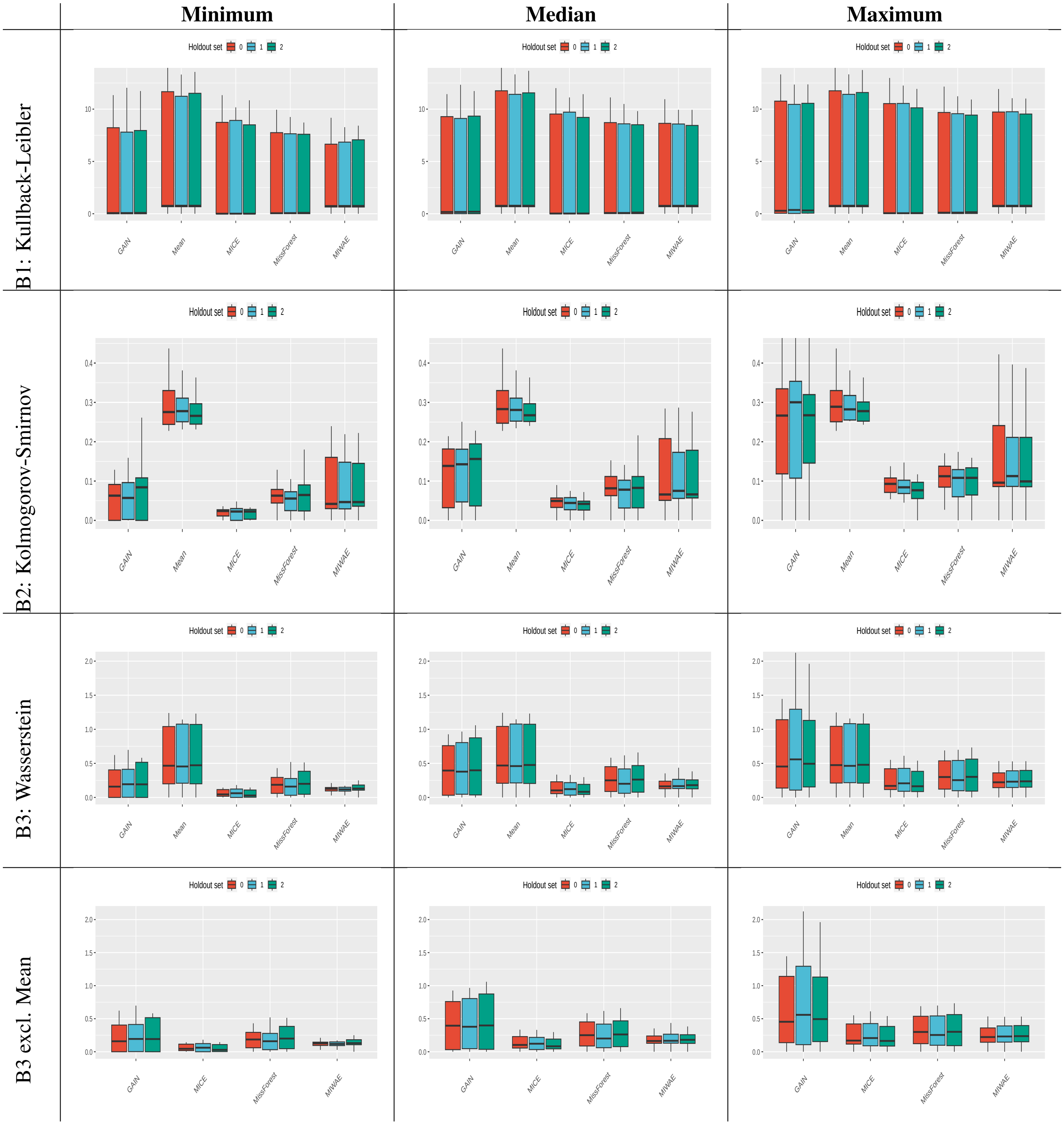

Supplementary Figure 23. Feature-wise 50% train missingness and 50% test missingness. Whiskers extend to the extreme values, with outliers omitted that are above 1.5 times the interquartile range from the median (horizontal line).

Sliced Wasserstein discrepancy for the MIMIC-III dataset at different train and test missingness rates

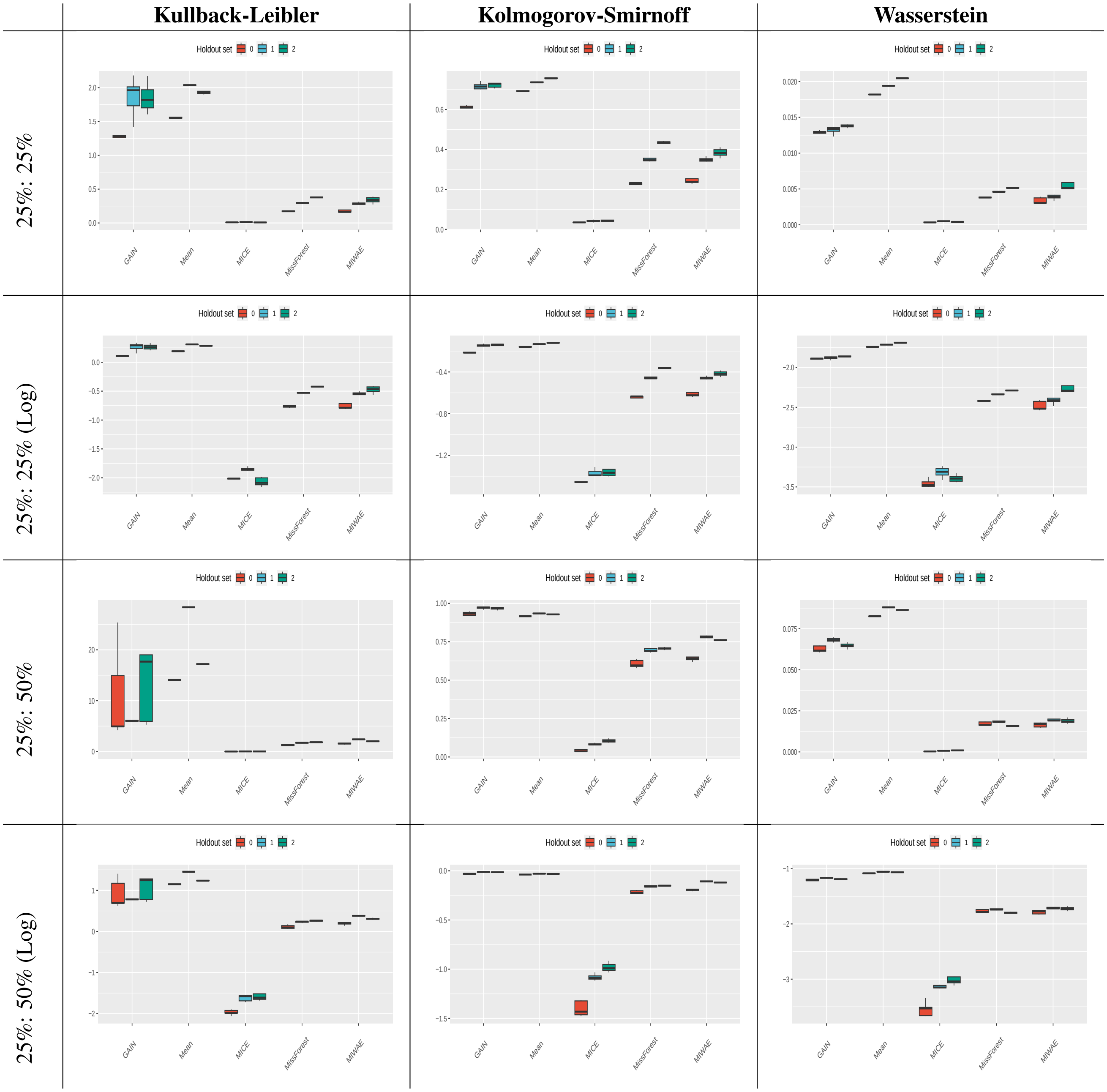

Supplementary Figure 24. The class C discrepancies for the sliced Wasserstein distances of the **MIMIC-III** data at the 25% missingness rate for the development set along with 25% and 50% for the test set. The original values and logarithms are shown for clarity. Whiskers extend to the extreme values, with outliers omitted that are above 1.5 times the interquartile range from the median (horizontal line).

Sliced Wasserstein discrepancy for the MIMIC-III dataset at different train and test missingness rates

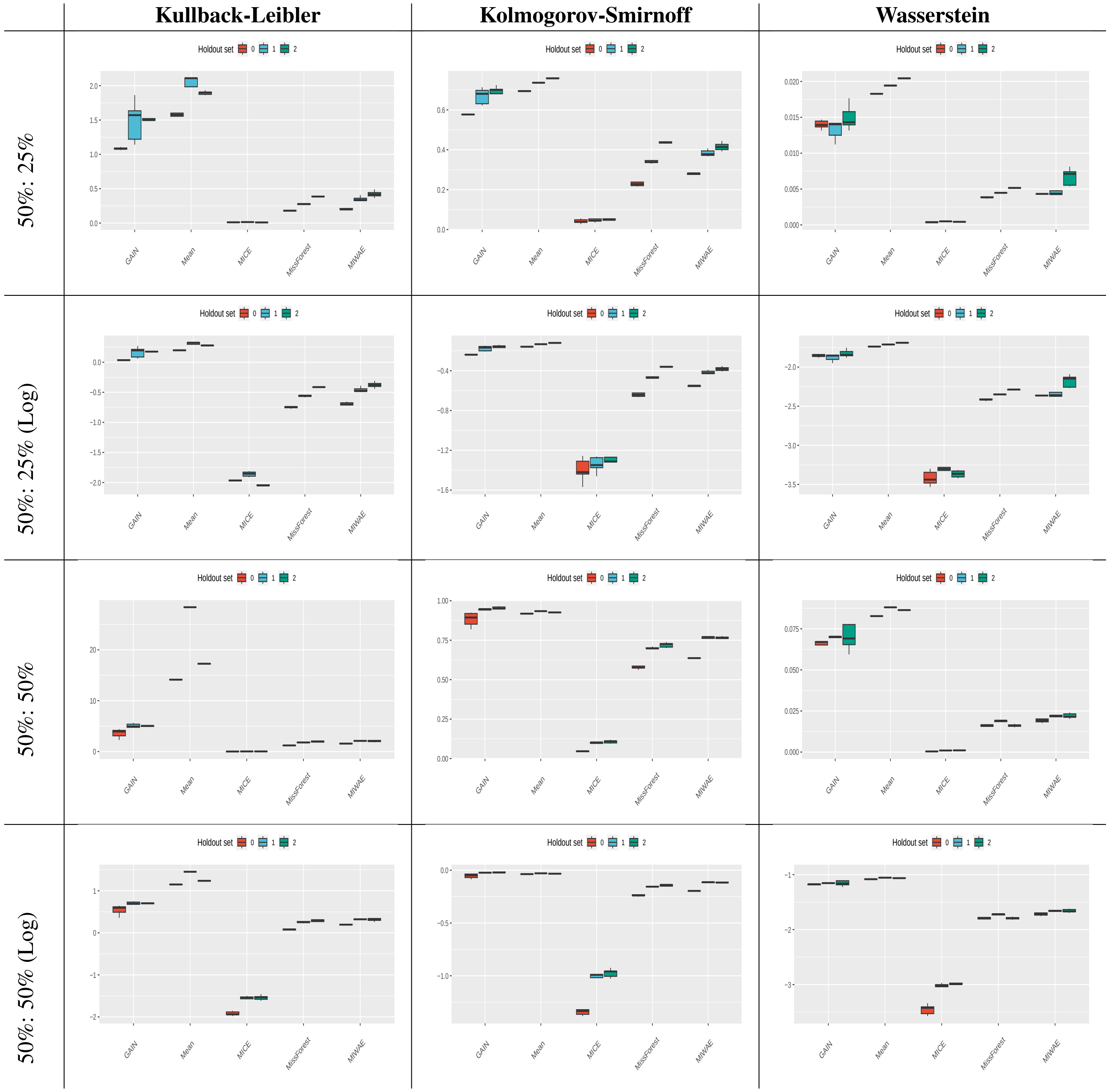

Supplementary Figure 25. The class C discrepancies for the sliced Wasserstein distances of the **MIMIC-III** data at the 50% missingness rate for the development set along with 25% and 50% for the test set. The original values and logarithms are shown for clarity. Whiskers extend to the extreme values, with outliers omitted that are above 1.5 times the interquartile range from the median (horizontal line).

Sliced Wasserstein discrepancy for the Simulated (N) dataset at different train and test missingness rates

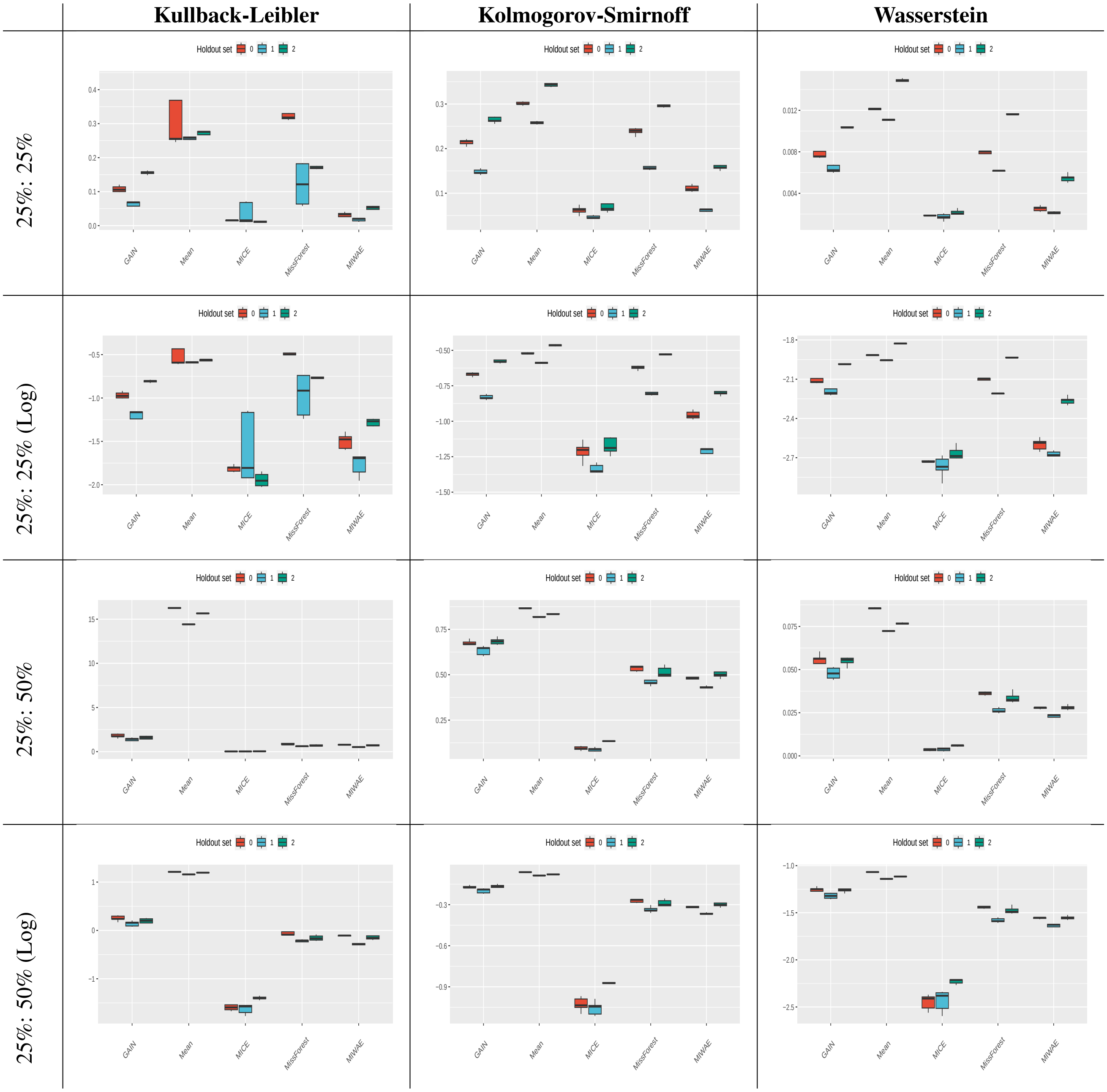

Supplementary Figure 26. The class C discrepancies for the sliced Wasserstein distances of the **Simulated (N)** dataset with 25% missingness for the development set along with 25% and 50% for the test set. The original values and their logarithms are shown for clarity. Whiskers extend to the extreme values, with outliers omitted that are above 1.5 times the interquartile range from the median (horizontal line).

Sliced Wasserstein discrepancy for the Simulated (N) dataset at different train and test missingness rates

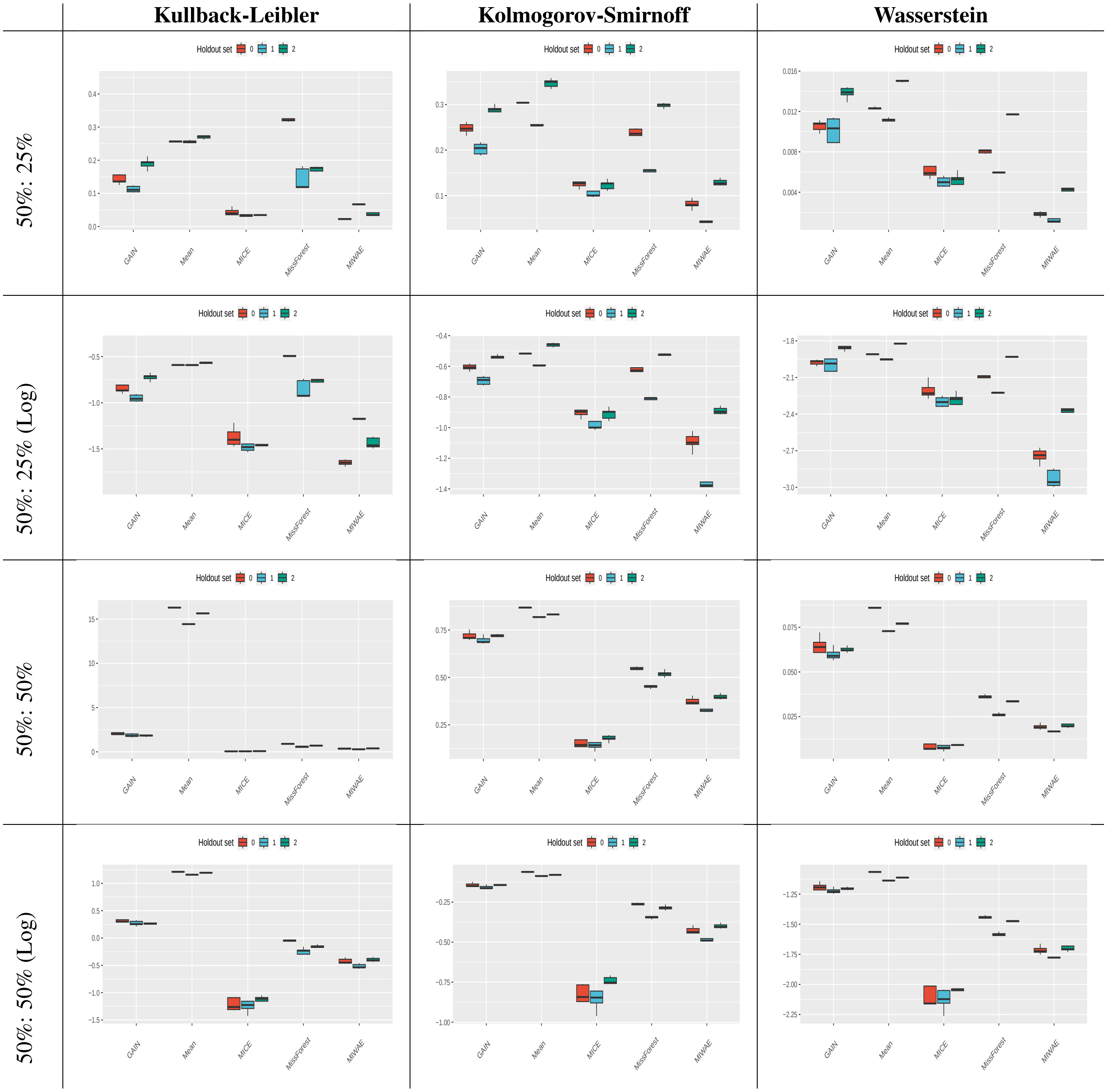

Supplementary Figure 27. The class C discrepancies for the sliced Wasserstein distances of the **Simulated (N)** data with 50% missingness for the development set along with 25% and 50% for the test set. The original values and their logarithms are shown for clarity. Whiskers extend to the extreme values, with outliers omitted that are above 1.5 times the interquartile range from the median (horizontal line).

Sliced Wasserstein discrepancy for the Simulated (N,C) dataset at different train and test missingness rates

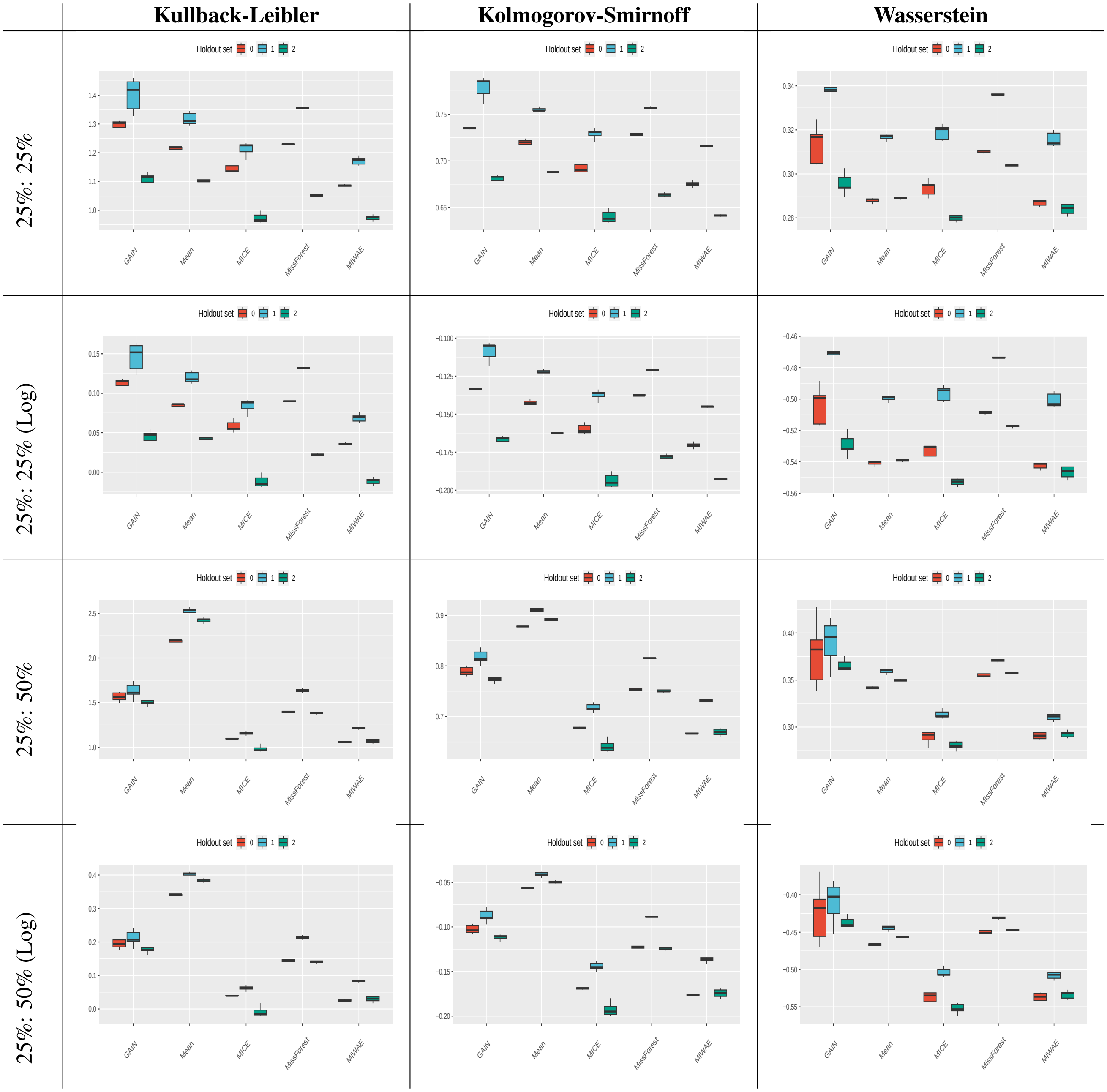

Supplementary Figure 28. The class C discrepancies for the sliced Wasserstein distances of the **Simulated (N,C)** data with 25% missingness for the development set along with 25% and 50% for the test set. The original values and their logarithms are shown for clarity. Whiskers extend to the extreme values, with outliers omitted that are above 1.5 times the interquartile range from the median (horizontal line).

Sliced Wasserstein discrepancy for the Simulated (N,C) dataset at different train and test missingness rates

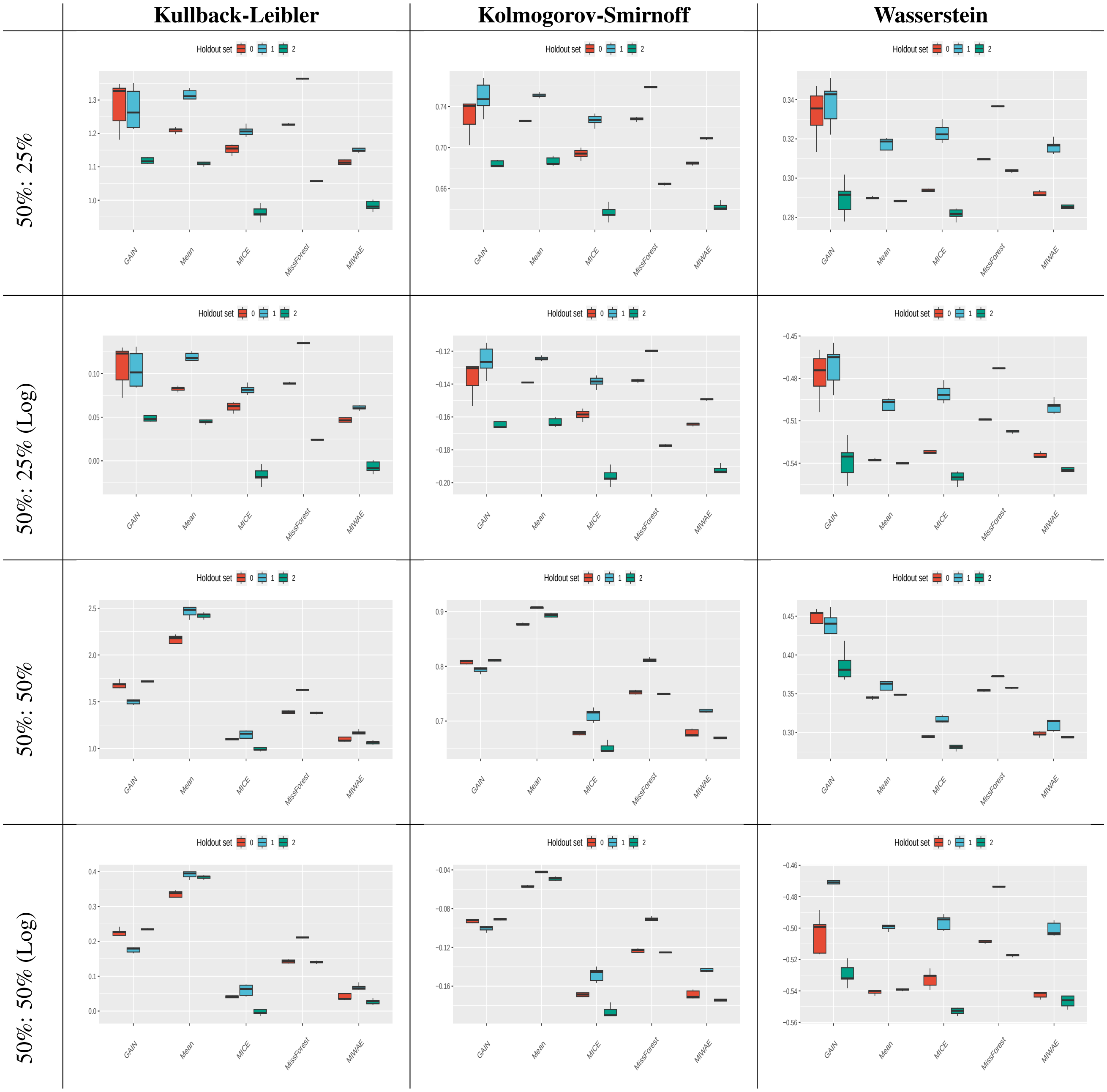

Supplementary Figure 29. The class C discrepancies for the sliced Wasserstein distances of the **Simulated (N,C)** data at the 50% missingness rate for the development set along with 25% and 50% for the test set. The original values and their logarithms are shown for clarity. Whiskers extend to the extreme values, with outliers omitted that are above 1.5 times the interquartile range from the median (horizontal line).

Link between quality and downstream classification performance

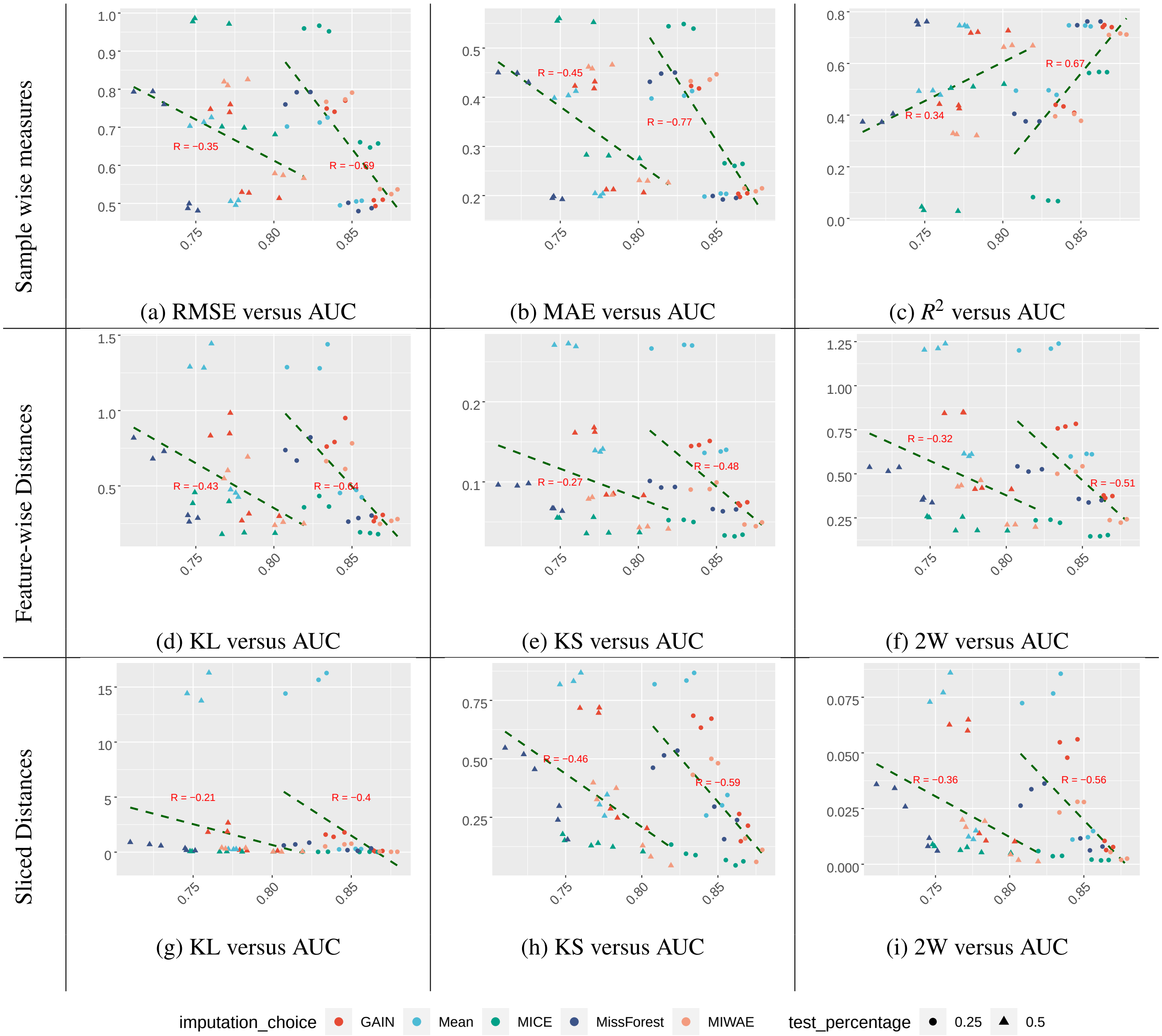

Supplementary Figure 30. The different imputation discrepancy metrics from classes A, B and C are shown against the downstream AUC value for the classification task of the **Simulated (N)** dataset. Trend lines are shown for 25% and 50% test missingness separately.

Link between quality and downstream classification performance

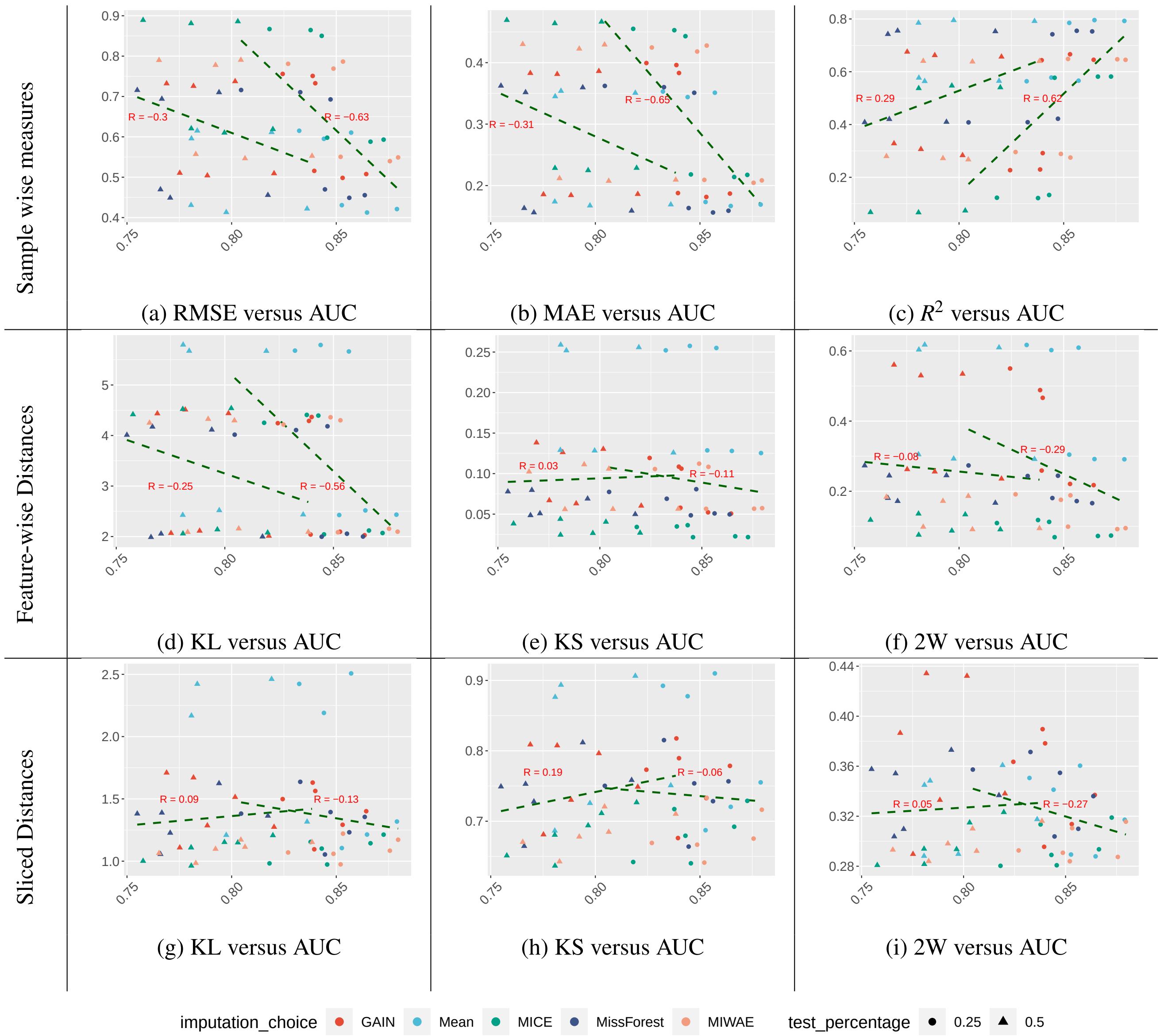

Supplementary Figure 31. The different imputation discrepancy metrics from classes A, B and C are shown against the downstream AUC value for the classification task of the **Simulated (N,C)** dataset. Trend lines are shown for 25% and 50% test missingness separately.

Additional Distance Ratio Figures

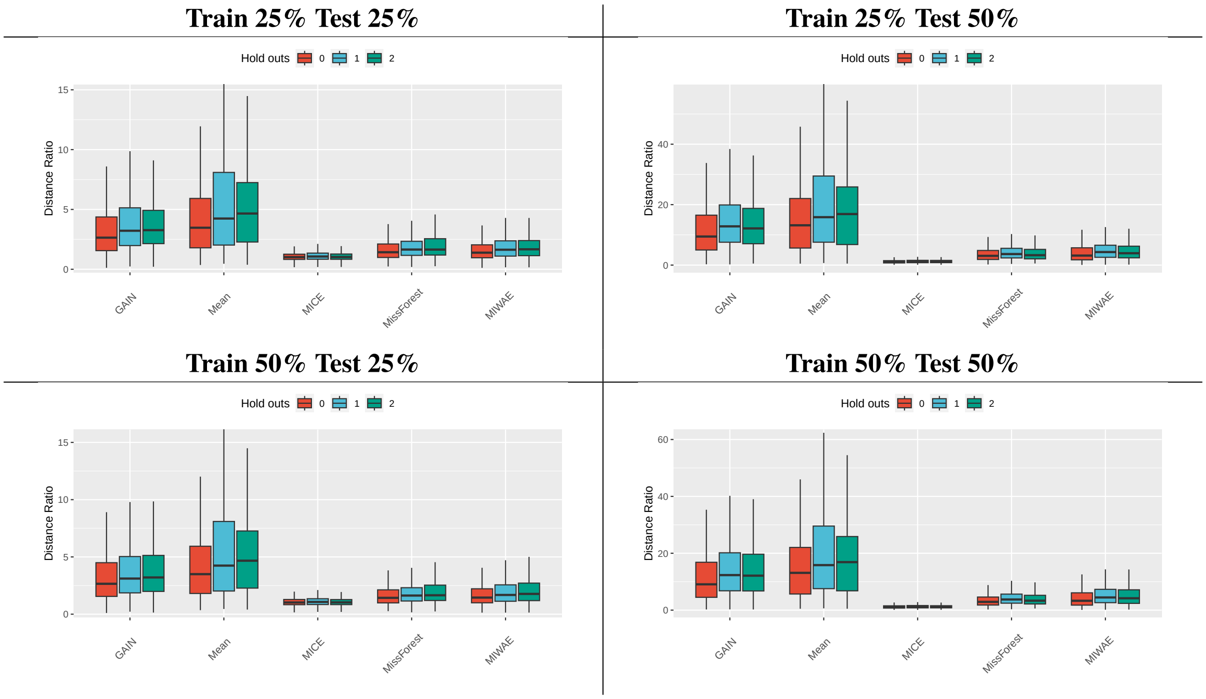

Supplementary Figure 32. Ratio of the Wasserstein distance for the imputed data compared to the original data for the **MIMIC-III** dataset at different train and test missingness rates. Whiskers extend to the extreme values, with outliers omitted that are above 1.5 times the interquartile range from the median (horizontal line).

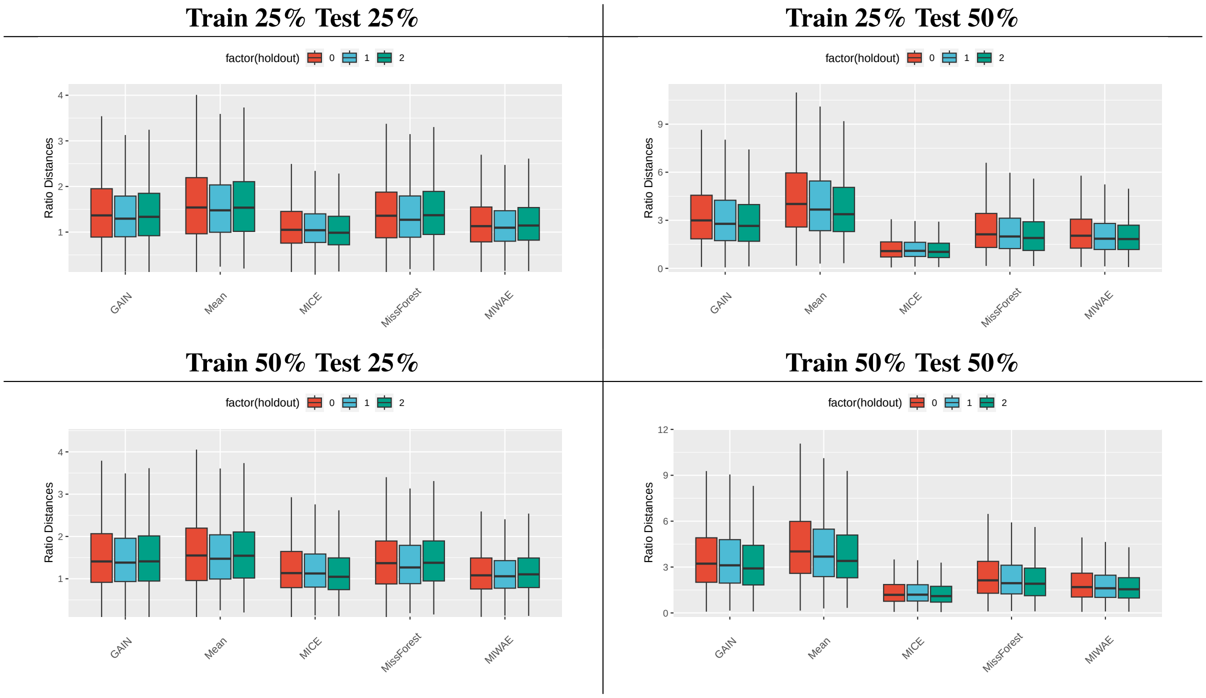

Supplementary Figure 33. Ratio of the Wasserstein distance for the imputed data compared to the original data for the **Simulated (N)** dataset at different train and test missingness rates. Whiskers extend to the extreme values, with outliers omitted that are above 1.5 times the interquartile range from the median (horizontal line).

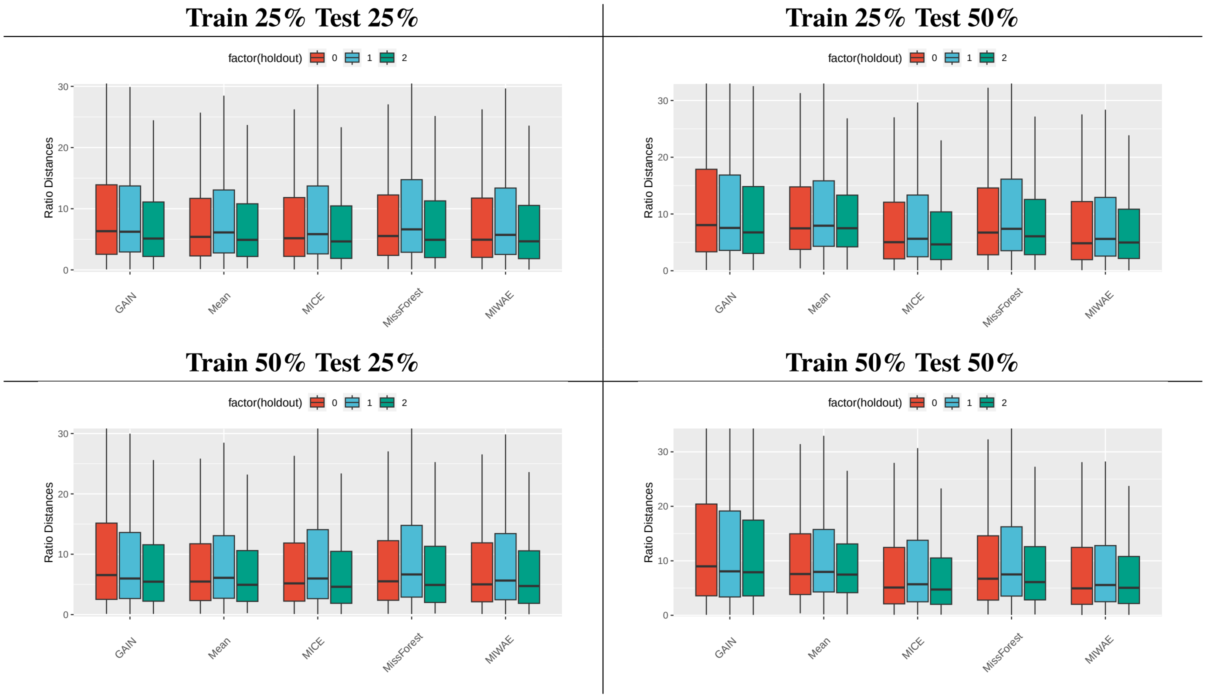

Supplementary Figure 34. Ratio of the Wasserstein distance for the imputed data compared to the original data for the **Simulated (N,C)** dataset at different train and test missingness rates. Whiskers extend to the extreme values, with outliers omitted that are above 1.5 times the interquartile range from the median (horizontal line).

Correlation between discrepancy statistics for the Simulated (N) and Simulated (N,C) datasets.

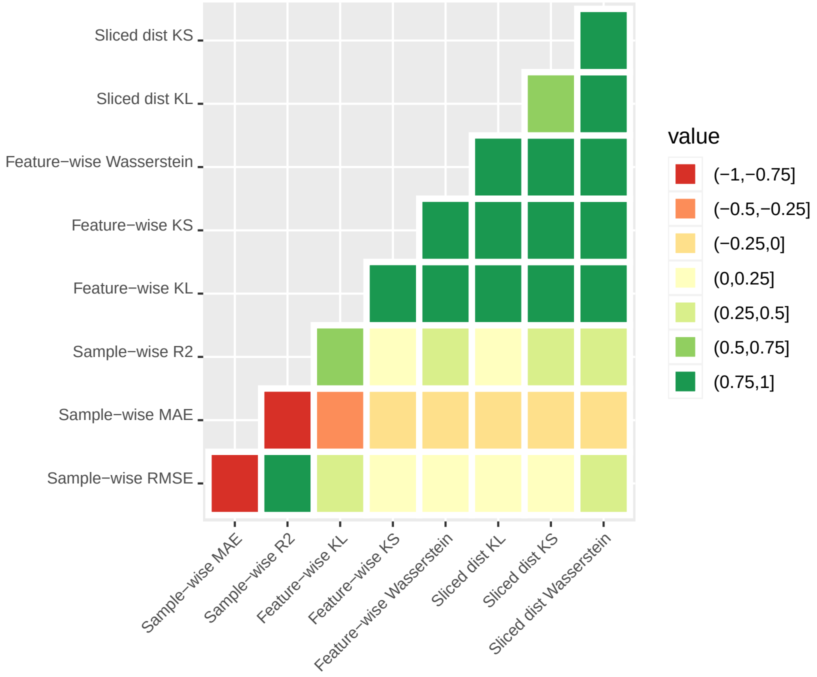

(a) Simulated (N) data set

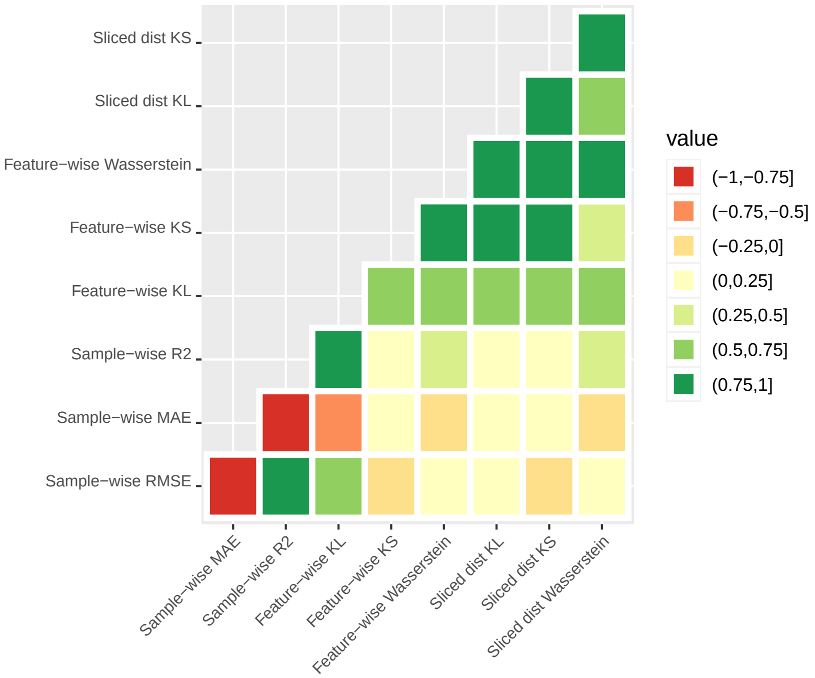

(b) Simulated (N,C) data set

Supplementary Figure 35. Correlation heatmap for all discrepancy metrics considered in this paper for (a) the **Simulated (N)** dataset and (b) the **Simulated (N,C)** dataset.

Codebase flowchart

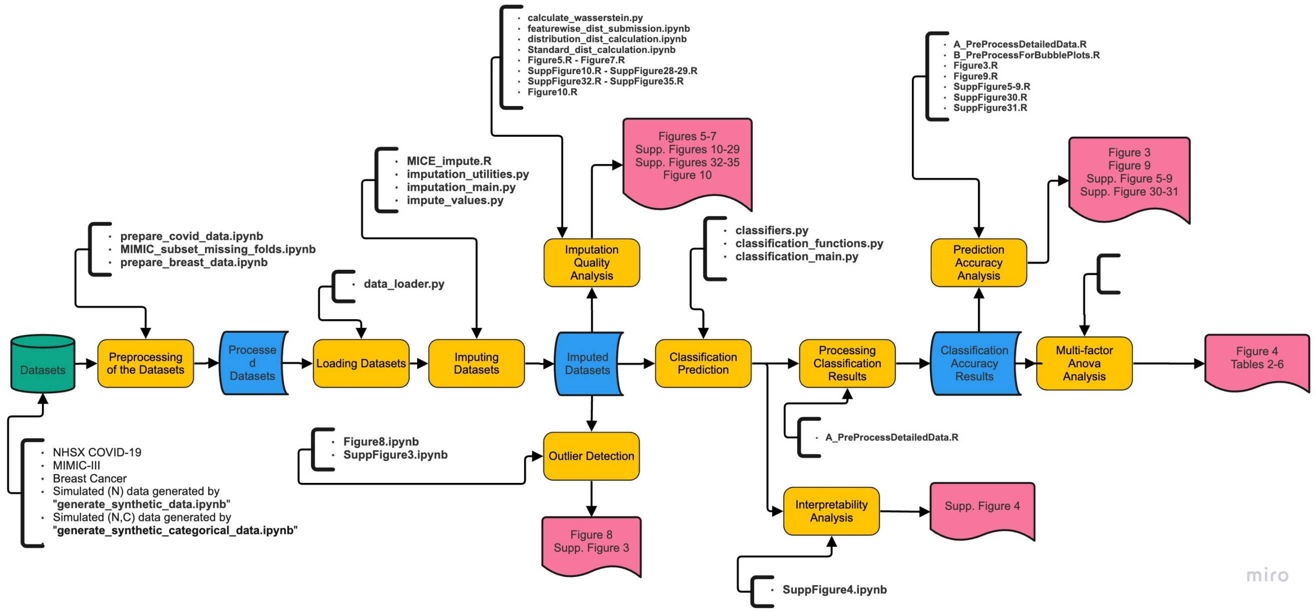

Supplementary Figure 36. Flowchart of the codebase. In yellow are shown the individual modules with the associated codes labeled. In pink, we indicate the Figures and Supplementary Figures generated at each step by these codes.

Supplementary Tables

ANOVA Results for the Breast Cancer, NHSX COVID-19 and MIMIC-III datasets

Variables	Estimate	Std. Error	t value	Pr($>|t|$)	
classifier choice LogisticReg	1.0824	0.0188	57.50	<2e-16	***
classifier choice NeuralNetwork	0.9841	0.0184	53.52	<2e-16	***
classifier choice NGBoost	1.0046	0.0185	54.37	<2e-16	***
classifier choice RandomForest	1.0475	0.0187	56.12	<2e-16	***
classifier choice XGBoost	1.0419	0.0186	55.90	<2e-16	***

Supplementary Table 2. Table of parameter coefficients for the **Breast Cancer** dataset.

Variables	Estimate	Std. Error	t value	Pr($>|t|$)	
imputation choice GAIN	1.1323	0.0340	33.33	<2e-16	***
imputation choice Mean	1.1689	0.0342	34.22	<2e-16	***
imputation choice MICE	1.1299	0.0340	33.27	<2e-16	***
imputation choice MissForest	1.0688	0.0337	31.74	<2e-16	***
imputation choice MIWAE	1.1764	0.0342	34.40	<2e-16	***
classifier choice Neural Network	-0.0959	0.0354	-2.71	0.0085	**
classifier choice NGBoost	0.0357	0.0360	0.99	0.3239	
classifier choice Random Forest	0.0305	0.0359	0.85	0.3989	
classifier choice XGBoost	0.0749	0.0361	2.07	0.0423	*

Supplementary Table 3. Table of parameter coefficients for the **NHSX COVID-19** dataset

Variables	Estimate	Std. Error	t value	Pr($>|t|$)	
imputation choice GAIN	1.0530	0.0130	81.03	<2e-16	***
imputation choice Mean	1.0119	0.0129	78.21	<2e-16	***
imputation choice MICE	1.0721	0.0130	82.33	<2e-16	***
imputation choice MissForest	1.0467	0.0130	80.60	<2e-16	***
imputation choice MIWAE	1.0661	0.0130	81.92	<2e-16	***
classifier choice Neural Network	0.0237	0.0123	1.93	0.0548	.
classifier choice NGBoost	0.0963	0.0124	7.78	1.3e-13	***
classifier choice RandomForest	0.0610	0.0123	4.94	1.3e-06	***
classifier choice XGBoost	0.1321	0.0124	10.62	<2e-16	***
train percentage 0.5	-0.0175	0.0079	-2.23	0.0268	*
test percentage 0.5	-0.1531	0.0079	-19.47	<2e-16	***

Supplementary Table 4. Table of parameter coefficients for the **MIMIC-III** dataset.

ANOVA results for the Simulated (N) dataset

Variables	Estimate	Std. Error	t value	Pr(> $\|t\|$)	
imputation choice GAIN	1.6777	0.0306	54.79	<2e-16	***
imputation choice Mean	1.6191	0.0302	53.61	<2e-16	***
imputation choice MICE	1.6253	0.0302	53.74	<2e-16	***
imputation choice MissForest	1.6552	0.0302	54.85	<2e-16	***
imputation choice MIWAE	1.7848	0.0313	57.01	<2e-16	***
classifier choice Neural Network	0.9962	0.0459	21.69	<2e-16	***
classifier choice NGBoost	-0.0190	0.0387	-0.49	0.6241	
classifier choice Random Forest	-0.0482	0.0385	-1.25	0.2112	
classifier choice XGBoost	0.2913	0.0404	7.22	6.31e-12	***
train percentage 0.5	-0.1057	0.0330	-3.21	0.0015	**
test percentage 0.5	-0.4593	0.0320	-14.34	<2e-16	***
imputation choice Mean:train percentage 0.5	0.0130	0.0269	0.48	0.6285	
imputation choice MICE:train percentage 0.5	-0.0301	0.0271	-1.11	0.2675	
imputation choice MissForest:train percentage 0.5	-0.0334	0.0266	-1.26	0.2105	
imputation choice MIWAE:train percentage 0.5	-0.0671	0.0276	-2.43	0.0157	*
imputation choice Mean:classifier choice Neural Network	-0.0534	0.0456	-1.17	0.2429	
imputation choice MICE:classifier choice Neural Network	-0.0842	0.0453	-1.86	0.0642	.
imputation choice MissForest:classifier choice Neural Network	-0.2190	0.0445	-4.92	1.58e-06	***
imputation choice MIWAE:classifier choice Neural Network	-0.0732	0.0464	-1.58	0.1159	
imputation choice Mean:classifier choice NGBoost	-0.0507	0.0410	-1.23	0.2182	
imputation choice MICE:classifier choice NGBoost	0.0408	0.0411	0.99	0.3226	
imputation choice MissForest:classifier choice NGBoost	-0.0666	0.0407	-1.64	0.1030	
imputation choice MIWAE:classifier choice NGBoost	-0.0090	0.0419	-0.22	0.8298	
imputation choice Mean:classifier choice RandomForest	-0.1012	0.0408	-2.48	0.0137	*
imputation choice MICE:classifier choice RandomForest	0.0109	0.0409	0.27	0.7907	
imputation choice MissForest:classifier choice RandomForest	-0.1162	0.0405	-2.87	0.0044	**
imputation choice MIWAE:classifier choice RandomForest	-0.0416	0.0417	-1.00	0.3196	
imputation choice Mean:classifier choice XGBoost	-0.1364	0.0420	-3.25	0.0013	**
imputation choice MICE:classifier choice XGBoost	0.0125	0.0423	0.29	0.7684	
imputation choice MissForest:classifier choice XGBoost	-0.1325	0.0417	-3.18	0.0017	**
imputation choice MIWAE:classifier choice XGBoost	-0.0124	0.0432	-0.29	0.7743	
classifier choice NeuralNetwork:train percentage 0.5	-0.4091	0.0466	-8.79	2.40e-16	***
classifier choice NGBoost:train percentage 0.5	-0.0428	0.0394	-1.09	0.2786	
classifier choice RandomForest:train percentage 0.5	-0.0728	0.0389	-1.87	0.0626	.
classifier choice XGBoost:train percentage 0.5	-0.1845	0.0409	-4.52	9.70e-06	***
imputation choice Mean:test percentage 0.5	0.0415	0.0272	1.52	0.1286	
imputation choice MICE:test percentage 0.5	0.0186	0.0274	0.68	0.4966	
imputation choice MissForest:test percentage 0.5	-0.0984	0.0269	-3.65	0.0003	***
imputation choice MIWAE:test percentage 0.5	0.0305	0.0279	1.09	0.2753	
classifier choice Neural Network:test percentage 0.5	-0.5158	0.0442	-11.67	<2e-16	***
classifier choice NGBoost:test percentage 0.5	-0.0235	0.0378	-0.62	0.5335	
classifier choice RandomForest:test percentage 0.5	0.0170	0.0375	0.45	0.6513	
classifier choice XGBoost:test percentage 0.5	-0.1445	0.0392	-3.69	0.0003	***
train percentage 0.5:test percentage 0.5	0.0569	0.0374	1.52	0.1292	
classifier choice Neural Network:train percentage 0.5:test percentage 0.5	0.1876	0.0591	3.17	0.0017	**
classifier choice NGBoost:train percentage 0.5:test percentage 0.5	0.0169	0.0524	0.32	0.7468	
classifier choice RandomForest:train percentage 0.5:test percentage 0.5	0.0260	0.0520	0.50	0.6172	
classifier choice XGBoost:train percentage 0.5:test percentage 0.5	0.0778	0.0539	1.44	0.1501	

Supplementary Table 5. Table of parameter coefficients for **Simulated (N)** dataset.

ANOVA results for the Simulated (N,C) dataset

Variables	Estimate	Std. Error	t value	Pr(> $\|t\|$)	
imputation choice GAIN	1.6643	0.0330	50.48	<2e-16	***
imputation choice Mean	1.7395	0.0332	52.45	<2e-16	***
imputation choice MICE	1.6787	0.0330	50.86	<2e-16	***
imputation choice MissForest	1.6214	0.0329	49.33	<2e-16	***
imputation choice MIWAE	1.7386	0.0332	52.43	<2e-16	***
classifier choice NeuralNetwork	0.2492	0.0419	5.95	8.02e-09	***
classifier choice NGBoost	0.1131	0.0411	2.75	0.0062	**
classifier choice RandomForest	0.1018	0.0410	2.48	0.0136	*
classifier choice XGBoost	0.2143	0.0417	5.13	5.30e-07	***
train percentage 0.5	-0.1369	0.0352	-3.89	0.0001	***
test percentage 0.5	-0.3711	0.0349	-10.64	<2e-16	***
classifier choice NeuralNetwork:train percentage 0.5	-0.1274	0.0452	-2.82	0.0052	**
classifier choice NGBoost:train percentage 0.5	-0.0125	0.0448	-0.28	0.7814	
classifier choice RandomForest:train percentage 0.5	-0.0465	0.0448	-1.04	0.3001	
classifier choice XGBoost:train percentage 0.5	-0.0766	0.0453	-1.69	0.0923	.
classifier choice NeuralNetwork:test percentage 0.5	-0.1441	0.0455	-3.16	0.0017	**
classifier choice NGBoost:test percentage 0.5	-0.0877	0.0451	-1.94	0.0529	.
classifier choice RandomForest:test percentage 0.5	-0.0488	0.0450	-1.08	0.2793	
classifier choice XGBoost:test percentage 0.5	-0.0954	0.0456	-2.09	0.0373	*
train percentage 0.5:test percentage 0.5	0.0994	0.0290	3.43	0.0007	***

Supplementary Table 6. Table of parameter coefficients for **Simulated (N,C)** dataset.

Configurations of the models used in the interpretability analysis

Classifier	Imputation	Train Missing-ness	Test Missing-ness	Holdout Set	Distance Ratio	Mean Test AUC	Max Depth	Min Samples Split	Min Samples Leaf	Estimators
Random Forest	MICE	0.25	0.25	1	1.16	0.83	4	2	4	60
Random Forest	Mean	0.25	0.25	0	1.68	0.83	4	2	3	90

Supplementary Table 7. The model configurations used in the interpretability analysis for the Random Forest.

Classifier	Imputation	Train Missing-ness	Test Missing-ness	Holdout Set	Distance Ratio	Mean Test AUC	Max Depth	Estimators	Subsampling
XGBoost	MICE	0.25	0.25	2	1.11	0.87	5	400	0.6
XGBoost	GAIN	0.25	0.25	0	1.51	0.88	5	400	0.6

Supplementary Table 8. The model configurations used in the interpretability analysis for XGBoost.

Classifier	Imputation	Train Missing-ness	Test Missing-ness	Holdout Set	Distance Ratio	Mean Test AUC	Estimators	Learning Rate	Minibatch Fraction
NGBoost	MICE	0.25	0.25	1	1.16	0.84	400	0.01	0.5
NGBoost	GAIN	0.25	0.25	0	1.51	0.85	350	0.01	0.5

Supplementary Table 9. The model configurations used in the interpretability analysis for NGBoost.

AIX-COVNET

Michael Roberts[2,3], Sören Dittmer[2,15], Ian Selby[6], Anna Breger[2,16], Matthew Thorpe[4], Julian Gilbey[2], Jonathan R. Weir-McCall[6,17], Effrossyni Gkrania-Klotsas[8], Anna Korhonen[18], Emily Jefferson[19], Georg Langs[20], Guang Yang[21], Helmut Prosch[20], Jacobus Preller[8], Jan Stanczuk[2], Jing Tang[1], Judith Babar[8], Lorena Escudero Sánchez[6], Philip Teare[3], Mishal Patel[3,7], Marcel Wassin[22], Markus Holzer[22], Nicholas Walton[23], Pietro Lió[5], Tolou Shadbahr[1], James H. F. Rudd[10], Evis Sala[6] and Carola-Bibiane Schönlieb[2].

[16] Faculty of Mathematics, University of Vienna, Austria [17] Royal Papworth Hospital, Cambridge, Royal Papworth Hospital NHS Foundation Trust, Cambridge, UK [18] Language Technology Laboratory, University of Cambridge, Cambridge, UK. [19] Population Health and Genomics, School of Medicine, University of Dundee, Dundee, UK. [20] Department of Biomedical Imaging and Image-guided Therapy, Computational Imaging Research Lab Medical University of Vienna, Vienna, Austria. [21] National Heart and Lung Institute, Imperial College London, London, UK. [22] contextflow GmbH, Vienna, Austria. [23] Institute of Astronomy, University of Cambridge, Cambridge, UK.

Supplementary References

1. Johnson, A. E. W. *et al.* MIMIC-III, a freely accessible critical care database. *Sci. Data* **3**, 160035, DOI: 10.1038/sdata.2016.35 (2016).
2. Wang, S. *et al.* MIMIC-Extract: A Data Extraction, Preprocessing, and Representation Pipeline for MIMIC-III. *Proc. ACM Conf. on Heal. Inference, Learn.* 222–235, DOI: 10.1145/3368555.3384469 (2020).
3. Razavi, P. *et al.* The Genomic Landscape of Endocrine-Resistant Advanced Breast Cancers. *Cancer Cell* **34**, 427–438.e6, DOI: 10.1016/j.ccell.2018.08.008 (2018).
4. Pedregosa, F. *et al.* Scikit-learn: Machine Learning in Python. *J. Mach. Learn. Res.* **12**, 2825–2830 (2011).
5. Stekhoven, D. J. & Bühlmann, P. MissForest—non-parametric missing value imputation for mixed-type data. *Bioinformatics* **28**, 112–118, DOI: 10.1093/bioinformatics/btr597 (2012).
6. Breiman, L. Random Forests. *Mach. Learn.* **45**, 5–32, DOI: 10.1023/A:1010933404324 (2001).
7. Choudhury, A. & Kosorok, M. R. Missing Data Imputation for Classification Problems. *arXiv* DOI: 10.48550/arXiv.2002.10709 (2020).
8. Bhattarai, A. missingpy (2018). Https://github.com/epsilon-machine/missingpy version 0.2.0.
9. van Buuren, S. Multiple imputation of discrete and continuous data by fully conditional specification. *Stat. Methods Med. Res.* **16**, 219–242, DOI: 10.1177/0962280206074463 (2007).
10. Raghunathan, T. E., Lepkowski, J. M., Van Hoewyk, J. & Solenberger, P. A Multivariate Technique for Multiply Imputing Missing Values Using a Sequence of Regression Models. *Surv. Methodol.* **27**, 85–95 (2001).
11. van Buuren, S. & Oudshoorn, K. Flexible Multivariate Imputation by MICE. Tech. Rep. PG/VGZ/99.054, Netherlands Organization for Applied Scientific Research (TNO), Leiden, The Netherlands (1999).
12. Enders, C. K., Mistler, S. A. & Keller, B. T. Multilevel multiple imputation: A review and evaluation of joint modeling and chained equations imputation. *Psychol. Methods* **21**, 222–240, DOI: 10.1037/met0000063 (2016).
13. Ding, Y. & Ross, A. A comparison of imputation methods for handling missing scores in biometric fusion. *Pattern Recognit.* **45**, 919–933, DOI: 10.1016/j.patcog.2011.08.002 (2012).
14. White, I. R., Royston, P. & Wood, A. M. Multiple imputation using chained equations: Issues and guidance for practice. *Stat. Medicine* **30**, 377–399, DOI: 10.1002/sim.4067 (2011).
15. Tran, C. T., Zhang, M., Andreae, P., Xue, B. & Bui, L. T. An effective and efficient approach to classification with incomplete data. *Knowledge-Based Syst.* **154**, 1–16, DOI: 10.1016/j.knosys.2018.05.013 (2018).
16. van Buuren, S. & Groothuis-Oudshoorn, K. mice: Multivariate Imputation by Chained Equations in R. *J. Stat. Softw.* **45**, 1–67, DOI: 10.18637/jss.v045.i03 (2011).
17. Yoon, J., Jordon, J. & van der Schaar, M. GAIN: Missing Data Imputation using Generative Adversarial Nets. In *Proceedings of the 35th International Conference on Machine Learning*, vol. 80 of *Proceedings of Machine Learning Research*, 5689–5698 (PMLR, 2018).
18. Goodfellow, I. *et al.* Generative Adversarial Nets. In *Advances in Neural Information Processing Systems*, vol. 27 (Curran Associates, Inc., 2014).

19. Wang, Z., Akande, O., Poulos, J. & Li, F. Are deep learning models superior for missing data imputation in large surveys? Evidence from an empirical comparison. *arXiv* DOI: 10.48550/arXiv.2103.09316 (2022).

20. Mattei, P.-A. & Frellsen, J. MIWAE: Deep Generative Modelling and Imputation of Incomplete Data Sets. In *Proceedings of the 36th International Conference on Machine Learning*, vol. 97 of *Proceedings of Machine Learning Research*, 4413–4423 (PMLR, 2019).

21. Kingma, D. P. & Welling, M. Auto-Encoding Variational Bayes. In *2nd International Conference on Learning Representations, ICLR 2014*, DOI: 10.48550/arXiv.1312.6114 (Banff, AB, Canada, 2014).

22. Rezende, D. J., Mohamed, S. & Wierstra, D. Stochastic Backpropagation and Approximate Inference in Deep Generative Models. In *Proceedings of the 31st International Conference on Machine Learning*, 1278–1286 (PMLR, 2014).

23. Burda, Y., Grosse, R. & Salakhutdinov, R. Importance Weighted Autoencoders. *arXiv* DOI: 10.48550/arXiv.1509.00519 (2016).

24. Cox, D. R. The regression analysis of binary sequences. *J. Royal Stat. Soc. Ser. B (Methodological)* **20**, 215–232, DOI: 10.1111/j.2517-6161.1958.tb00292.x (1958). _eprint: https://onlinelibrary.wiley.com/doi/pdf/10.1111/j.2517-6161.1958.tb00292.x.

25. Breiman, L. Consistency for a simple model of random forests. Technical Report 670, Statistics Department, University of California at Berkeley (2004).

26. Chen, T. & Guestrin, C. XGBoost: A Scalable Tree Boosting System. In *Proceedings of the 22nd ACM SIGKDD International Conference on Knowledge Discovery and Data Mining*, KDD '16, 785–794, DOI: 10.1145/2939672.2939785 (Association for Computing Machinery, New York, NY, USA, 2016).

27. Duan, T. *et al.* NGBoost: natural gradient boosting for probabilistic prediction. In *Proceedings of the 37th International Conference on Machine Learning*, Proceedings of Machine Learning Research, 2690–2700 (PMLR, 2020).

28. Martens, J. New Insights and Perspectives on the Natural Gradient Method. *J. Mach. Learn. Res.* **21**, 1–76 (2020).

29. Hastie, T., Tibshirani, R. & Friedman, J. *The Elements of Statistical Learning: Data Mining, Inference, and Prediction*. Springer Series in Statistics (Springer, New York, NY, USA, 2009), 2nd edn.

30. Rumelhart, D. E., Hinton, G. E. & Williams, R. J. Learning representations by back-propagating errors. *Nature* **323**, 533–536, DOI: 10.1038/323533a0 (1986).

31. Agarap, A. F. Deep Learning using Rectified Linear Units (ReLU). *arXiv* DOI: 10.48550/arXiv.1803.08375 (2019).

32. Kingma, D. P. & Ba, J. Adam: A Method for Stochastic Optimization. In *3rd International Conference on Learning Representations (ICLR)* (San Diego, CA, USA, 2015).

33. Villani, C. *Optimal Transport: Old and New*. Grundlehren der mathematischen Wissenschaften (Springer Berlin, Heidelberg, 2009), 1st edn.

34. Arjovsky, M., Chintala, S. & Bottou, L. Wasserstein Generative Adversarial Networks. In *Proceedings of the 34th International Conference on Machine Learning*, 214–223 (PMLR, 2017).

35. Lundberg, S. M. & Lee, S.-I. A Unified Approach to Interpreting Model Predictions. In *Advances in Neural Information Processing Systems*, vol. 30 (Curran Associates, Inc., 2017).

36. Van Rossum, G. & Drake, F. L. *Python 3 Reference Manual* (CreateSpace, Scotts Valley, CA, 2009).

37. R Core Team. *R: A Language and Environment for Statistical Computing*. R Foundation for Statistical Computing, Vienna, Austria (2021).

38. van Buuren, S. & Groothuis-Oudshoorn, K. mice: Multivariate imputation by chained equations in r. *J. Stat. Softw.* **45**, 1–67, DOI: 10.18637/jss.v045.i03 (2011).
