# Supplementary material for: The impact of imputation quality on machine learning classifiers for datasets with missing values
Source: Commun Med (Lond). 2023 Oct 6;3:139. doi: 10.1038/s43856-023-00356-z (PMC10558448; doi:10.1038/s43856-023-00356-z)
Supplement: Supplementary file 3 — Reporting Summary [file 43856_2023_356_MOESM3_ESM.pdf]

nature research

Corresponding author(s): Michael Roberts
Last updated by author(s): Aug 10, 2023

Reporting Summary

Nature Research wishes to improve the reproducibility of the work that we publish. This form provides structure for consistency and transparency in reporting. For further information on Nature Research policies, see our Editorial Policies and the Editorial Policy Checklist.

Statistics

For all statistical analyses, confirm that the following items are present in the figure legend, table legend, main text, or Methods section.

n/a	Confirmed	
☐	☒	The exact sample size (n) for each experimental group/condition, given as a discrete number and unit of measurement
☐	☒	A statement on whether measurements were taken from distinct samples or whether the same sample was measured repeatedly
☐	☒	The statistical test(s) used AND whether they are one- or two-sided<br>*Only common tests should be described solely by name; describe more complex techniques in the Methods section.*
☐	☒	A description of all covariates tested
☐	☒	A description of any assumptions or corrections, such as tests of normality and adjustment for multiple comparisons
☐	☒	A full description of the statistical parameters including central tendency (e.g. means) or other basic estimates (e.g. regression coefficient) AND variation (e.g. standard deviation) or associated estimates of uncertainty (e.g. confidence intervals)
☐	☒	For null hypothesis testing, the test statistic (e.g. F, t, r) with confidence intervals, effect sizes, degrees of freedom and P value noted<br>*Give P values as exact values whenever suitable.*
☒	☐	For Bayesian analysis, information on the choice of priors and Markov chain Monte Carlo settings
☐	☒	For hierarchical and complex designs, identification of the appropriate level for tests and full reporting of outcomes
☒	☐	Estimates of effect sizes (e.g. Cohen's d, Pearson's r), indicating how they were calculated

Our web collection on statistics for biologists contains articles on many of the points above.

Software and code

Policy information about availability of computer code

Data collection	Three of the datasets (MIMIC, Breast Cancer and NHSX COVID-19) were collected by other institutes and available on reasonable request to the data access committee of the relevant originator. For the Synthetic datasets, we generated this using a Python package as detailed in the manuscript.
Data analysis	For the preprocessing of the Breast Cancer dataset, we use our implementation provided in the code prepare_breast_data.ipynb as a Jupyter notebook by using python 3.6. For the preprocessing of a random subset of MIMIC-III dataset the code MIMIC_subset_missing_folds.ipynb is used. A full description of the preprocessing steps are included in our Supplementary Materials for both Breast Cancer and MIMIC-III datasets. The NHSX COVID-19 dataset is preprocessed by code provided in the prepare_covid_data.ipynb Jupyter notebook. All the packages and software used in these analysis are open source and publicly available.

For manuscripts utilizing custom algorithms or software that are central to the research but not yet described in published literature, software must be made available to editors and reviewers. We strongly encourage code deposition in a community repository (e.g. GitHub). See the Nature Research guidelines for submitting code & software for further information.

Data

Policy information about availability of data

All manuscripts must include a data availability statement. This statement should provide the following information, where applicable:

- Accession codes, unique identifiers, or web links for publicly available datasets
- A list of figures that have associated raw data
- A description of any restrictions on data availability

The data that support the findings of this study are openly available for the simulated, MIMIC-III and Breast Cancer datasets in our GitLab repository (10.5281/zenodo.8234032). The NHSX COVID-19 dataset is available from the originators upon completion of an approved request (see https://nhsx.github.io/covid-chest-

imaging-database/data-access). All code required to pre-process the data is shared in our repository.

The MIMIC-III dataset is available after completion of a training course at https://physionet.org/content/mimic3-carevue/1.4/. The Breast Cancer dataset is freely available at https://www.cbioportal.org/study/summary?id=breast_msk_2018.

Field-specific reporting

Please select the one below that is the best fit for your research. If you are not sure, read the appropriate sections before making your selection.

☒ Life sciences ☐ Behavioural & social sciences ☐ Ecological, evolutionary & environmental sciences

For a reference copy of the document with all sections, see nature.com/documents/nr-reporting-summary-flat.pdf

Life sciences study design

All studies must disclose on these points even when the disclosure is negative.

Sample size	Inherent to the data collected from the different sources for the clinical datasets. For the Synthetic (N) data, a sample size of 1000 was chosen, as with 25 features an event per variable of 40 gives a low risk of bias in the data and for a predictive model based on the PROBAST guidelines. The Synthetic (N,C) dataset has 100 samples with 20 informative features and 5 useless features -- again with a low risk of bias per PROBAST.
Data exclusions	For the clinical datasets NHSX COVID-19 and Breast Cancer, features were removed that were >50% incomplete and samples were removed that were >50% incomplete to ensure there was sufficient data to perform reasonable imputation. The Synthetic dataset is complete by design. For the MIMIC-III dataset, all incomplete records are excluded from the beginning as the data is of sufficient scale.
Replication	We have been careful to ensure that the data processing, imputation, classifiers and analysis are completely reproducible.
Randomization	N/A
Blinding	N/A

Reporting for specific materials, systems and methods

We require information from authors about some types of materials, experimental systems and methods used in many studies. Here, indicate whether each material, system or method listed is relevant to your study. If you are not sure if a list item applies to your research, read the appropriate section before selecting a response.

Materials & experimental systems

n/a	Involved in the study	
☒	☐	Antibodies
☒	☐	Eukaryotic cell lines
☒	☐	Palaeontology and archaeology
☒	☐	Animals and other organisms
☒	☐	Human research participants
☐	☒	Clinical data
☒	☐	Dual use research of concern

Methods

n/a	Involved in the study	
☒	☐	ChIP-seq
☒	☐	Flow cytometry
☒	☐	MRI-based neuroimaging

Clinical data

Policy information about clinical studies

All manuscripts should comply with the ICMJE guidelines for publication of clinical research and a completed CONSORT checklist must be included with all submissions.

Clinical trial registration	N/A
Study protocol	N/A
Data collection	We use existing clinical datasets, which are referenced in the manuscript and data collection methodology has been detailed at source.
Outcomes	The outcomes are inherent to the datasets. Mortality is the outcome of interest for all clinical datasets.
